# The PICLS high-throughput screening method for agents extending cellular longevity identifies 2,5-anhydro-D-mannitol as novel anti-aging compound

**DOI:** 10.1007/s11357-022-00598-0

**Published:** 2022-06-15

**Authors:** Mohammad Alfatah, Frank Eisenhaber

**Affiliations:** 1 Bioinformatics Institute (BII), Singapore, 138671 A*STAR Singapore; 2 Genome Institute of Singapore (GIS), Singapore, 138672 A*STAR Singapore; 3 School of Biological Sciences (SBS), Nanyang Technological University (NTU), Singapore, 637551 Singapore

**Keywords:** *Saccharomyces cerevisiae*, Chronological lifespan, Chemical screening, Anti-aging compound, 2,5-anhydro-D-mannitol

## Abstract

Although aging is the biggest risk factor for human chronic (cancer, diabetic, cardiovascular, and neurodegenerative) diseases, few interventions are known besides caloric restriction and a small number of drugs (with substantial side effects) that directly address aging. Thus, there is an urgent need for new options that can generally delay aging processes and prevent age-related diseases. Cellular aging is at the basis of aging processes. Chronological lifespan (CLS) of yeast *Saccharomyces cerevisiae* is the well-established model system for investigating the interventions of human post-mitotic cellular aging. CLS is defined as the number of days cells remain viable in a stationary phase. We developed a new, cheap, and fast quantitative method for measuring CLS in cell cultures incubated together with various chemical agents and controls on 96-well plates. Our PICLS protocol with (1) the use of propidium iodide for fluorescent-based cell survival reading in a microplate reader and (2) total cell count measurement via OD600nm absorption from the same plate provides real high-throughput capacity. Depending on logistics, large numbers of plates can be processed in parallel so that the screening of thousands of compounds becomes feasible in a short time. The method was validated by measuring the effect of rapamycin and calorie restriction on yeast CLS. We utilized this approach for chemical agent screening. We discovered the anti-aging/geroprotective potential of 2,5-anhydro-D-mannitol (2,5-AM) and suggest its usage individually or in combination with other anti-aging interventions.

**Supplementary Information:**

The online version contains supplementary material available at 10.1007/s11357-022-00598-0.

## Introduction

The growing fraction of the elderly (> 65 years) among the total population (from ~ 10% worldwide (above 20% in first world countries) in 2020 to extrapolated 16% in 2050), as well as their increasing life expectancy (by ~ 10 years since 1960 in first world countries), confronts the world with increasingly difficult problems [1, 2]. Besides generally reducing life activity, aging remains the greatest risk factor for the development of chronic diseases that, subsequently, compromise independent human life and finally lead to death [3–7]. The current medical approaches to prevent age-related pathologies are recommendations for a healthy lifestyle, including exercise and diet. However, these interventions alone are not sufficient to prevent the onset of age-related diseases.

Aging is characterized by a progressive loss of physiological integrity, efficiency in cellular functions, and metabolic signaling [8]. Increasing efforts are directed to understand cellular aging processes affecting the highly interconnected and functionally redundant gene and protein interactions network [8, 9]. Despite the complexity of aging, recent research in different model systems, including mammals, has demonstrated that delayed aging and increased healthspan are feasible by anti-aging interventions such as rapamycin drug administration application and calorie (glucose) restriction [10–15]. Therefore, expanding the repertoire of anti-aging compounds that can be utilized as geroprotecting therapeutics and reducing their unwanted side effects is one of the promising strategies that can delay aging and prolong health span.

Recent research has shown that molecular mechanisms observed in human aging are conserved in different organisms, including single-cell yeast [8, 10, 16, 17]. The budding yeast *Saccharomyces cerevisiae* is one of the most studied model organisms for uncovering the biological processes involved in cellular aging. The benefits of studying aging in yeast include a short generation time, a tractable lifespan, and its amenability to high-throughput assays. Therefore, this organism became a powerful tool for the identification of anti-aging interventions. Yeast is commonly used to study aging in two distinct ways: replicative lifespan (RLS) and chronological lifespan (CLS) [18]. The RLS measures the number of times an individual cell divides, an aging model for mitotic cells such as stem cells. The CLS measures the length of time a non-dividing cell remains viable in the stationary phase, an aging model for post-mitotic cells such as neurons. The viability of aging yeast cells under nutrient-deprived stationary phase conditions decreases, and they eventually die.

CLS has been traditionally measured by estimating colony-forming units (CFUs) counts on the agar plate [18]. Yeast cells in a flask with total media (≥ 20 ml) are exposed to a single chemical compound. At different time points (e.g., after 2, 5, and 8 days), small aliquots of the chronological aging culture from the flask are serially diluted, plated onto nutrient agar plates, and incubated for 2–3 days for colonies formation and counting. The survival fraction determines from the CFU for each chronological age-point relative to day 1 (considered 100% cell survival). The CFU approach is quantitative but associated with many downsides. It requires multiple serial dilutions, plating steps, and incubation periods for appearing the colonies. Colony counting is performed either by manual or expensive instruments. Moreover, this method is unsuitable and much too expensive for high-throughput screening (HTS) of thousands of compounds because it requires substantial amounts of the drugs tested, large volume flask cultures, and many agar plates. Unfortunately, the CFU method and all recent variants for measuring CLS are critically dependent on the ability of the post-mitotic cells to re-enter into the mitotic phase. In principle, this methodology cannot distinguish yeast in mitotic arrest from dead cells.

Current technical advancements in CFU-derived methods measure cell survival and growth based on the outgrowth of drug-exposed stationary phase yeast in nutrient-rich culture either in liquid medium or by a spotting assay on an agar plate [17, 19–21]. After 24 h, the outgrowth of aged cells in liquid medium is measured by the absorbance at OD600nm, whereas in the spotting assay, the outgrowth of spotted aged culture onto agar medium is visually identified after 48 h. Logistical improvement includes the usage of 96-well plates that allow limited chemical or genome-wide deletion strains screening. For pooled tagged yeast deletion strains, flow cytometry is required to quantify the individual strain viability [22].

Propidium iodide (PI) is a fluorescent dye that only enters the dead cells and, thus, is a powerful marker for direct quantification of cell viability [23–25]. Previously, studies utilized a PI-based approach for measuring CLS and quantifying cell viability by analyzing the images acquired by a fluorescent cell counter, UV transilluminator, or flow cytometry [26, 27]. However, the multiple sample processing, imaging, and software requirements for data analysis are expensive and time-consuming, which hindered these methods in real high-throughput applications. The introduction of alternative dyes such as SYTOX green is of limited value in a high-throughput setting because of the prohibitive costs [26].

Therefore, new methods are required to quantify the viability of the aged cell to avoid the limitations associated with existing methodologies. In this work, we describe PICLS, a PI-based CLS measurement method that is outgrowth-independent. Our new approach relies on incubating yeast cells with various test compounds or differing test media conditions in 96-well plates. We use PI to quantify cell survival. Fast and efficient readout (within ~ 15 min) is provided by a microplate reader, a low-cost device readily available in most laboratories. This cheap methodology is well suited for large-scale screening of the chemical agents to identify anti-aging compounds as many different compounds, controls, or dilutions can be conveniently placed on a single plate and large numbers of plates can be processed in parallel. Furthermore, we validated our method by determining the extension of CLS of yeast using known anti-aging interventions such as rapamycin administration and calorie restriction. A quick screen of available chemicals in our laboratory unexpectedly revealed 2,5-anhydro-D-mannitol (2,5-AM) extending the lifespan of yeast. We also confirmed the anti-aging activity of 2,5-AM with traditional outgrowth methods. Other tested sugar analogs did not deliver any similar effect.

## Results

### Development of a method for quantification of cell viability using propidium iodide fluorescence measurement in the microplate reader

Our new protocol starts with exposing wild-type yeast cells or their mutants placed into nutrient-rich media in 96-well plates together with a specific concentration of chemical compounds of interest. We use PI as a marker for cell death as this fluorescent dye only enters non-surviving cells [23–25]. The cell viability in the stationary phase culture is quantified after a specified number of days by reading the PI fluorescence in the microplate reader that, in turn, provides an electronically readable spreadsheet for data analysis.

**Fig. 1:**
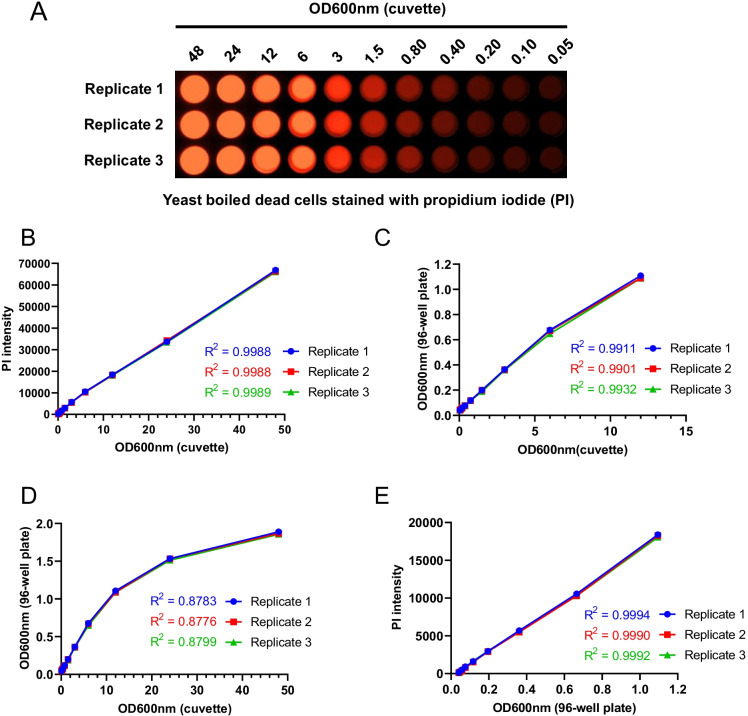
Development of propidium iodide fluorescence–based method for measuring the chronological lifespan. The propidium iodide (PI) fluorescence–based method was developed to measure the CLS of the yeast for screening the chemical agents to identify the anti-aging compounds. **A** Yeast boiled cells were stained with PI for 15 min at 30 °C. PI-stained cells were diluted (48 to 0.05 OD600nm) in a black 96-well plate and pseudo imaged with a BioRad GelDoc imaging system. Three replicates of each PI-stained sample were imaged and are represented in the figure. **B** Correlation between PI fluorescence intensity and cell OD600nm of the cuvette. **C** and **D** Correlation between cell OD600nm of the 96-well plate and the cuvette. **E** Correlation between PI fluorescence intensity and cell OD600nm of 96-well plate

As a first validation, we show that working with small amounts in microplate wells delivers results similar to cuvette-based approaches and that the microplate reader is sufficiently sensitive to capture cell survival data. Thus, we analyzed the PI staining of yeast cells. Yeast cells were grown in the glass flask and boiled for 15 min at 100 °C. Live and dead cells were washed and incubated in 1 × PBS (phosphate-buffered saline) without and with PI (5 µg/ml) for 15 min. After incubation, cells were washed and resuspended in PBS. Cells were visualized by microscopy and fluorescence intensity was measured by the microplate reader (Supplementary Fig. S1). After confirmation that PI is suitable marker for cell death, a subsequent analysis was performed for developing the method. PI-stained dead cells were resuspended in PBS to the final 48 OD600nm measured in the cuvette using a spectrophotometer. Next, cells were serially diluted in PBS (OD600nm 48 to 0.05) and transferred to a black 96-well plate (costar 3603). To note, black microplates are four times more expensive than clear plates and less easily available, but we used them since they are more suitable for fluorescence readout due to dampening autofluorescence originating from the samples and microplate surfaces. With a GelDoc imaging system (Fig. 1A), we visualized the degree of incorporation of PI staining in the dead yeast cells at various dilutions. Using the same plate, we measured the PI fluorescence intensity with a microplate reader at excitation 535 nm and emission 617 nm wavelengths. We found a high linear correlation between PI fluorescence intensity and cellular absorbance at OD600nm (Fig. 1B). This result indicates that our variant of the PI fluorescence–based method is effective for quantifying cell viability.

As a next step, we determined the optimal range of cell density (quantified by OD600nm) so that cell density could be directly measured in 96-well plates instead of in cuvettes. Using the black microplates with dead yeast cells from the previous experiment, we determined the correlation of OD600nm measurements taken from the cuvettes and from the microplate directly. We found a high linear correlation between the two series of measurements in the OD600nm range 0.05 to 12 of the cuvette (Fig. 1C). However, the trend is no longer maintained for higher OD600nm values > 12 of the cuvette (Fig. 1D). Next, we determined the relationship between PI fluorescence intensity and cell OD600nm of the 96-well plate within the optimal OD600nm range 0.05–12. We observe an almost perfect linear correlation (Fig. 1E). Thus, our protocol represents a robust method for quantifying cell viability.

### Effect of microplate types on quantification of cell viability using propidium iodide fluorescence measurement in the microplate reader

**Fig. 2:**
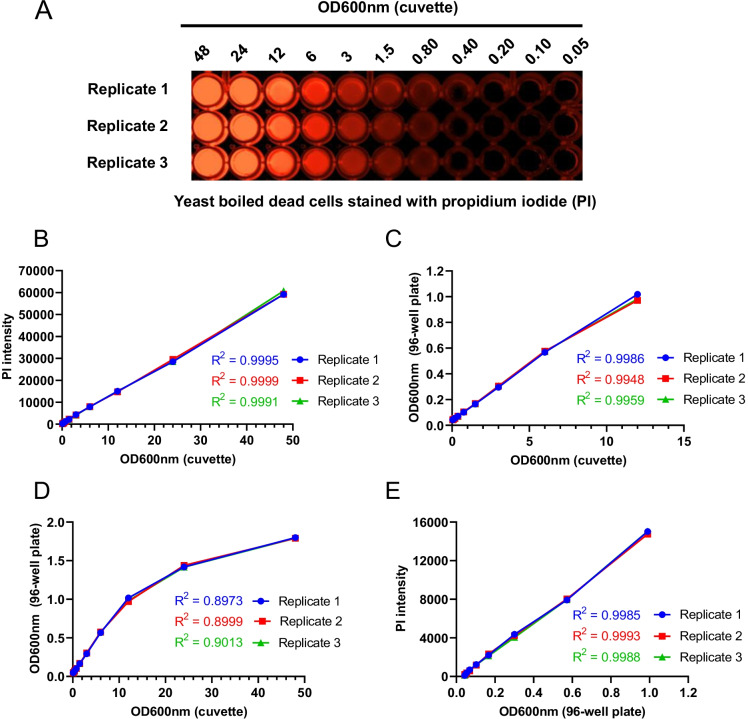
Determination of the propidium iodide fluorescence–based method for measuring the chronological lifespan in the clear 96-well plate. The propidium iodide (PI) fluorescence–based method for measuring the chronological lifespan (CLS) was determined in the clear 96-well plate for screening the chemical agents to identify the anti-aging compounds. **A** Yeast-boiled cells were stained with PI for 15 min at 30 °C. PI-stained cells were diluted (48 to 0.05 OD600nm) in a clear 96-well plate and pseudo imaged with a BioRad GelDoc imaging system. Three replicates of each PI-stained sample were imaged and are represented in the figure. **B** Correlation between PI fluorescence intensity and cell OD600nm of the cuvette. **C** and **D** Correlation between cell OD600nm of the clear 96-well plate and the cuvette. **E** Correlation between PI fluorescence intensity and cell OD600nm of the clear 96-well plate

**Fig. 3:**
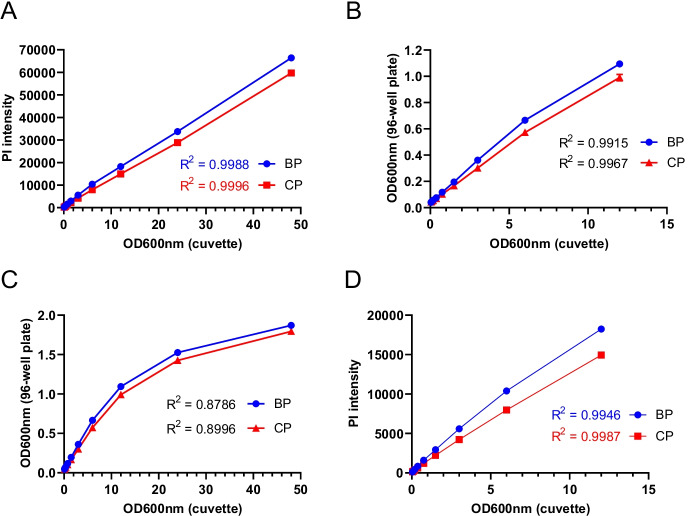
Comparison of the propidium iodide fluorescence–based method for measuring the chronological lifespan in black vs clear 96-well plates. **A** Comparison of black vs. clear 96-well plates’ correlation between PI fluorescence intensity and cell OD600nm of the cuvette. **B** and **C** Comparison of black vs. clear 96-well plates’ correlation between cell OD600nm of the 96-well plate and cuvette. **D** Comparison of black vs. clear 96-well plates’ correlation between PI fluorescence intensity and cell OD600nm of the 96-well plate

Though black microplates are recommended for fluorescence-based assays because they quench the background fluorescence, we wish to explore whether they can be substituted by the cheaper and easier available clear ones. We performed the experiments with dead yeast cells described above both in black and clear microplates (costar 3596). Sample staining was confirmed by visualizing the clear microplate using a GelDoc imaging system (Fig. 2A). After confirmation by imaging, we measured the PI fluorescence intensity using the microplate reader and determined the relationship with cell OD600nm values determined for the cuvette. We found a high linear correlation between PI fluorescence intensity and cell OD600nm intensity (Fig. 2B). We also obtained a high correlation between cell OD600nm values in the range 0.05 to 12 for the cuvette with the clear 96-well plate (Fig. 2C) and a distorted correlation for higher cell OD600nm values (> 12) of the cuvette (Fig. 2D). Furthermore, we also found a high linear correlation between PI fluorescence intensity and cell OD600nm intensity of the clear 96-well plate within the optimal range 0.05–12 (Fig. 2E). Finally, we compared the results between black and clear microplates. However, we did not observe any significant differences (Fig. 3A, B, C and D). Together, these results confirm that clear microplates are also suitable for our newly developed protocol for quantifying cell viability. Therefore, we can justifiably use clear microplates for all the subsequent experiments.

### Effect of rapamycin on the chronological lifespan of the yeast

Rapamycin is one of the well-known anti-aging interventions demonstrated to extend the lifespan of several model organisms, including yeast, nematodes, fruit flies, and mice [10, 14, 15, 28–30]. It inhibits the nutrient-sensing complex TORC1 (target of rapamycin complex 1). TORC1 is a conserved multi-subunit protein complex in eukaryotic cells that couples nutrients in the environment with cell growth and proliferation [19, 29, 31–34].

**Fig. 4:**
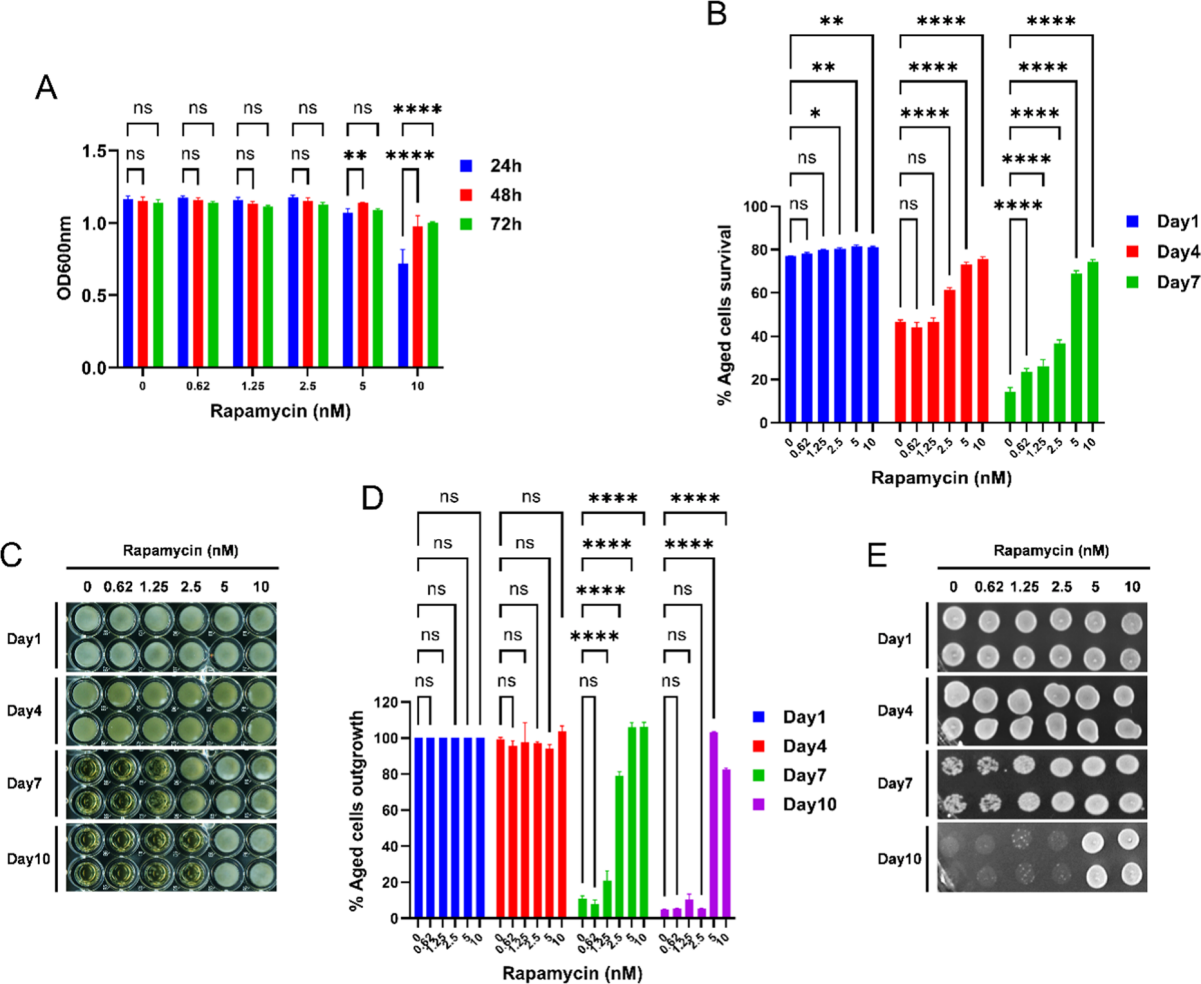
Rapamycin drug extends the chronological lifespan of the yeast. The prototrophic yeast strain (CEN.PK113-7D) was grown in the synthetic defined medium with different concentrations of rapamycin in 96-well plates at 30 °C. **A** Cell growth OD600nm was measured at time points 24 h, 48 h, and 72 h using a microplate reader and graph plotted against different concentrations of rapamycin. **B** The chronological lifespan (CLS) of different concentrations of rapamycin-incubated cells was determined using the propidium iodide fluorescence–based method. Cell survival at different chronological age points was quantified and the growth time point 72 h was considered as day 1. **C** The CLS of the aged cells was determined by the outgrowth method in YPD liquid medium. The growth time point 72 h was considered as day 1. At various chronological age points, a 3-μL culture was transferred to a second 96-well plate containing 200μL YPD medium. Outgrowth in 96-well plate was photographed after incubation for 24 h at 30 °C. **D** Outgrowth OD600nm in YPD liquid medium was measured using a microplate reader. The outgrowth of different chronological age points is plotted relative to day 1. **E** At various chronological age points, 3-μL cultures were spotted onto the YPD agar plate. Outgrowth was photographed after incubation for 48 h at 30 °C. All data represent as means ± SD.; *P < 0.05, **P < 0.01, and ****P < 0.0001 based on two-way ANOVA followed by Dunnett’s multiple comparisons test (**A**, **B**, and **D**). n.s, non-significant

We have examined the effect of rapamycin on the chronological lifespan (CLS) of the yeast strain to validate our newly developed protocol. Here and below, we used the prototrophic yeast strain (CEN.PK113-7D) in our experiments to avoid the strong effects of amino acid auxotrophy on cell survival in stationary phase culture [35, 36]. Cells were aged with different concentrations of rapamycin in the synthetic defined (SD) medium. Cell growth was measured at different time points (24 h, 48 h, and 72 h). We found that cell growth reached saturation approximately 24 h after incubating with 5 nM or less rapamycin (Fig. 4A). However, the administration of 10 nM rapamycin slowed cell growth, and saturation was reached only after 48 h (Fig. 4A). Previously, a similar trend was observed for cells growing with rapamycin in a CLS experiment [37].

As a next step, the CLS of cells grown in different concentrations of rapamycin was measured. We determined the cell survival using PI fluorescence at different chronological age time points. The growth time point 72 h was considered as day 1. We can deduce from the survival graph that different concentrations of rapamycin addition extend the CLS of yeast (Fig. 4B). The survival of aged cells supplemented with rapamycin (5 nM and 10 nM) on day 4 was ~ 75%; however, without rapamycin was ~ 50%. On day 7, survival of rapamycin supplemented aged cells was ~ 70%; however, without rapamycin was reduced to less than 20%. CLS extension of aged cells can also be observed at lower concentrations (0.62–2.5 nM) of rapamycin (Fig. 4B).

We have validated our protocol by directly comparing it with two traditional outgrowth approaches that measure the CLS of the yeast. Firstly, we conducted the outgrowth assay in the liquid medium to measure the effect of rapamycin on lifespan. The survival of chronological aging cells was monitored at different age points by transferring a 3-µl culture into 200μL of YPD medium in a fresh 96-well plate. Outgrowth corresponds to the number of viable cells in the inoculum (Fig. 4C). Aged cells outgrowth registered via OD600nm absorbance was determined using a microplate reader. The survival fraction was calculated from the outgrowth OD600nm absorbance for each age-point relative to day 1 (considered 100% cells survival). The survival graph is shown in Fig. 4D.

Likewise, we conducted the outgrowth by spotting assay on the agar medium. We spotted 3-µl aging culture onto the YPD agar plate and incubated it for 48 h. The outgrowth of aged cells on the YPD agar plate was visualized by the GelDoc imaging system (Fig. 4E). As expected, we can observe trends of lifespan extension by rapamycin under both YPD liquid and agar outgrowth assays that parallel those determined with our new protocol.

These results show that our HTS protocol reproduces that rapamycin extends the cellular lifespan. Furthermore, we assess the *Z*-factor to evaluate the quality of this method [38]. Generally, *Z*-factors in the range of 0.5–1.0 are indicative for excellent HTS assays. The *Z*-factor was determined for different concentrations of rapamycin with PI fluorescence of aged cells (Supplementary Fig. S2). Our finding, a *Z*-factor 0.70 for 5 nM rapamycin and 0.74 for 10 nM rapamycin, puts this HTS method into the high-quality category. Thus, our protocol is robust enough to identify potential anti-aging compounds.

For exploring whether the method is effective in a different yeast genetic background, we tested the effect of rapamycin on the CLS of BY4743 strain. *Saccharomyces cerevisiae* BY4743 strain is an auxotrophic for histidine, leucine, and uracil. The BY4743 strain was grown in different concentrations of rapamycin in the SD medium supplemented with auxotrophic constituents. Survival of aged cells was quantified using PI fluorescence (Supplementary Fig. S3). We found that rapamycin increased the viability of BY4743 cells quantitatively in similar ways as that of CEN.PK113-7D. Thus, these results reveal that our method effective in different yeast strains.

### Effect of glucose on the chronological lifespan of the yeast

**Fig. 5:**
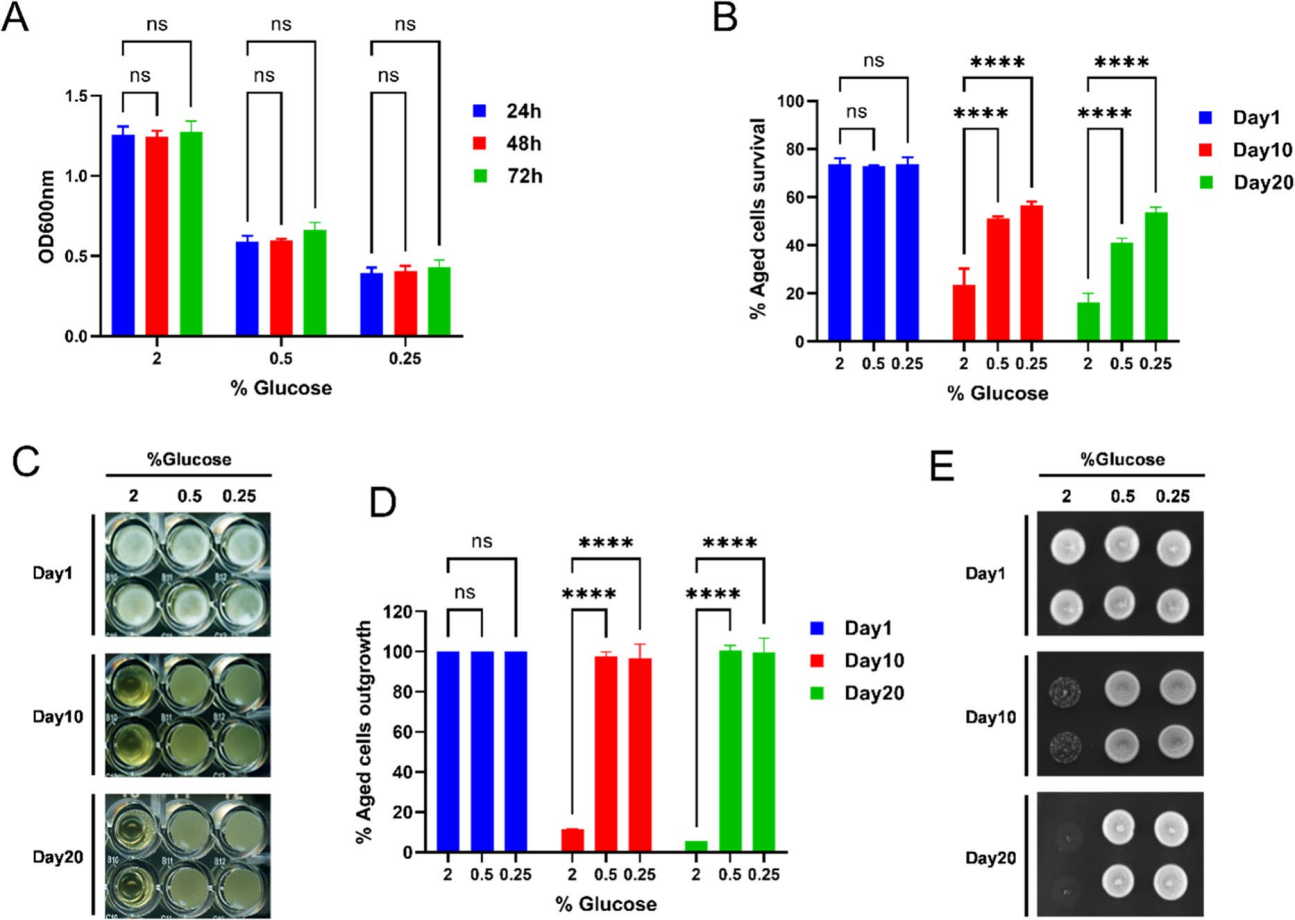
Calorie restriction extends the chronological lifespan of the yeast. The prototrophic yeast strain (CEN.PK113-7D) was grown in the synthetic defined medium containing 2%, 0.5%, and 0.25% glucose in 96-well plates at 30 °C. **A** Cell growth OD600nm was measured at time points 24 h, 48 h, and 72 h using a microplate reader and graph plotted against different glucose concentrations. **B** The chronological lifespan (CLS) of the aged cells grown under different glucose concentrations was determined using the propidium iodide fluorescence–based method. Cell survival at different chronological age points was quantified and the growth time point 72 h was considered as day 1. **C** The CLS of the aged cells grown under different glucose concentrations was determined by the outgrowth method in YPD liquid medium. The growth time point 72 h was considered as day 1. At various chronological age points, a 3-μL culture was transferred to a second 96-well plate containing 200μL YPD medium. Outgrowth in 96-well plate was photographed after incubation for 24 h at 30 °C. **D** Outgrowth OD600nm in YPD liquid medium was measured using a microplate reader. The graph is plotted relative to day 1. **E** At various chronological age points, 3-μL cultures were spotted onto the YPD agar plate. Outgrowth was photographed after incubation for 48 h at 30 °C. All data represent as means ± SD.; ****P < 0.0001 based on two-way ANOVA followed by Dunnett’s multiple comparisons test (**A**, **B**, and **D**). n.s, non-significant

We also validated our method by examining the effect of calorie restriction on the chronological lifespan of the yeast. Calorie restriction is one of the established interventions to delay aging and prolong the lifespan across the model organism, including yeast and mammalian cells [11, 13, 16]. To determine the effect of calorie restriction on the CLS of the yeast, we reduce the glucose content in the culture medium. We grew the cell in three different SD medium conditions containing 0.25%, 0.5%, and 2% glucose. We measured cell growth at different time points (24 h, 48 h, and 72 h). All cultures reached growth saturation after 24 h (Fig. 5A). Cell growth was lower in 0.25% and 0.5% compared to 2% glucose (Fig. 5A). We measured the survival of chronological aging for cells grown under different glucose concentrations using the PI fluorescence method (Fig. 5B). We infer from the survival graph that glucose restriction extends the CLS of the yeast. We also concurrently confirmed the extension of the CLS by outgrowth assays (Fig. 5C, D, and E), further validating our new method for determining the CLS of the yeast. The effect of glucose restriction on the CLS extension of the BY4743 strain was also shown by the PICLS method (Supplementary Fig. S3). Thus, our approach for measuring the CLS of the yeast represents a novel, fast and low-cost method for screening the chemical agents and culturing conditions to identify the anti-aging interventions.

#### Screening the chemical agents using the newly developed method identified 2,5-anhydro-D-mannitol as a novel anti-aging compound

**Fig. 6:**
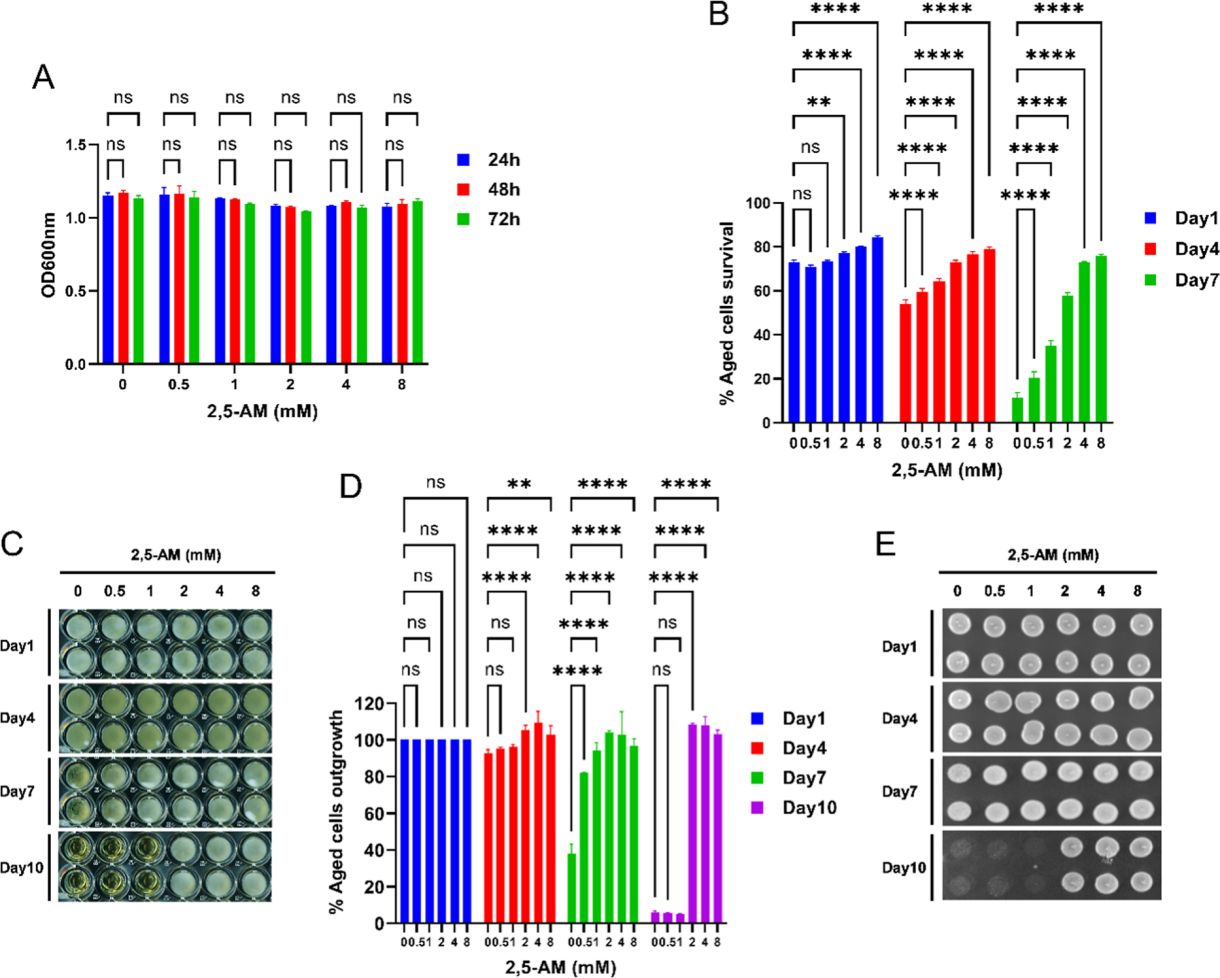
2,5-Anhydro-D-mannitol is a novel anti-aging compound that extends the chronological lifespan of the yeast. The prototrophic yeast strain (CEN.PK113-7D) was grown in the synthetic defined medium with different concentrations of 2,5-anhydro-D-mannitol (2,5-AM) in 96-well plates at 30 °C. **A** Cell growth OD600nm was measured at different time points 24 h, 48 h, and 72 h using a microplate reader and graph plotted against different 2,5-AM concentrations. **B** The chronological lifespan (CLS) of different concentrations of 2,5-AM incubated cells was determined using the propidium iodide fluorescence–based method. Cell survival at different chronological age points was quantified and the growth time point 72 h was considered as day 1. **C** The CLS of the aged cells was determined by the outgrowth method in YPD liquid medium. The growth time point 72 h was considered as day 1. At various chronological age points, 3-μL cultures were transferred to a second 96-well plate containing 200μL YPD medium. Outgrowth in 96-well plate was photographed after incubation for 24 h at 30 °C. **D** Outgrowth OD600nm in YPD liquid medium was measured using a microplate reader. The graph is plotted relative to day 1. **E** At various chronological age points, a 3-μL culture was spotted onto the YPD agar plate. Outgrowth was photographed after incubation for 48 h at 30 °C. All data represent as means ± SD.; **P < 0.01 and ****P < 0.0001 based on two-way ANOVA followed by Dunnett’s multiple comparisons test (**A**, **B**, and **D**). n.s, non-significant

After developing and validating the new protocol, we utilized this method to screen hundreds of chemical agents for the identification of anti-aging compounds (Supplementary Fig. S4 and Supplementary Table 1). As one of the surprising results, we found indications for the compound 2,5-anhydro-D-mannitol (2,5-AM) to extend the CLS of the yeast. In Fig. 6, we present the outcome of detailed follow-up experiments for this compound. We tested the anti-aging activity of 2,5-AM at different concentrations on the 96-well plate. Firstly, we determined the effect of 2,5-AM on cell growth. Yeast cells were incubated with varying concentrations of 2,5-AM in the SD medium. Cell growth was measured at different time points (24 h, 48 h, and 72 h). We found that cell growth reached the same saturation level after 24 h for all tested concentrations of 2,5-AM (Fig. 6A). Then, we measured the survival of chronologically aged cells supplemented with different concentrations of 2,5-AM using the PI fluorescence method. As previously, the 72h growth culture was considered day 1 for the CLS analysis. The survival graph is plotted for different chronological age time points (Fig. 6B). We find that 2,5-AM extends the CLS in a concentration-dependent manner. The survival of aged cells supplemented with 2,5-AM (8 mM) on day 4 was ~ 80%; however, without 2,5-AM was ~ 50%. On day 7, survival of 2,5-AM supplemented aged cells was ~ 75%; however, without 2,5-AM was reduced to less than 20%. The anti-aging activity of 2,5-AM was also verified by outgrowth assays (Fig. 6C, D, and E).

2,5-AM is a sugar molecule that can enter the glycolysis pathway. It gets hydrolyzed only at upstream glycolytic steps that cause accumulation of non-metabolized 2,5-AM-1,6-bisphosphate (2,5-AMBP) in the cells [39–41]. To test whether 2,5-AM at concentrations similar to the CLS experiment has been processed by glycolysis enzymes, we examined the growth sensitivity of the *SNF1* gene deletion strain. *SNF1* is a cellular energy sensor and highly conserved AMP-activated protein kinase (AMPK) in eukaryotes [42, 43]. Yeast cells that lack AMPK activity are associated with a hypersensitive growth phenotype in the presence of non-metabolized glycolytic intermediates [44]. We measured the growth of the *snf1Δ* deletion strain with different concentrations of 2,5-AM. We found that 2,5-AM inhibits the growth of *snf1Δ* deletion strain compared to wild-type strain (Supplementary Fig. S5). This observed growth phenotype indicates that 2,5-AM undergoes glycolysis and gets hydrolyzed into non-metabolized glycolytic intermediates.

**Fig. 7:**
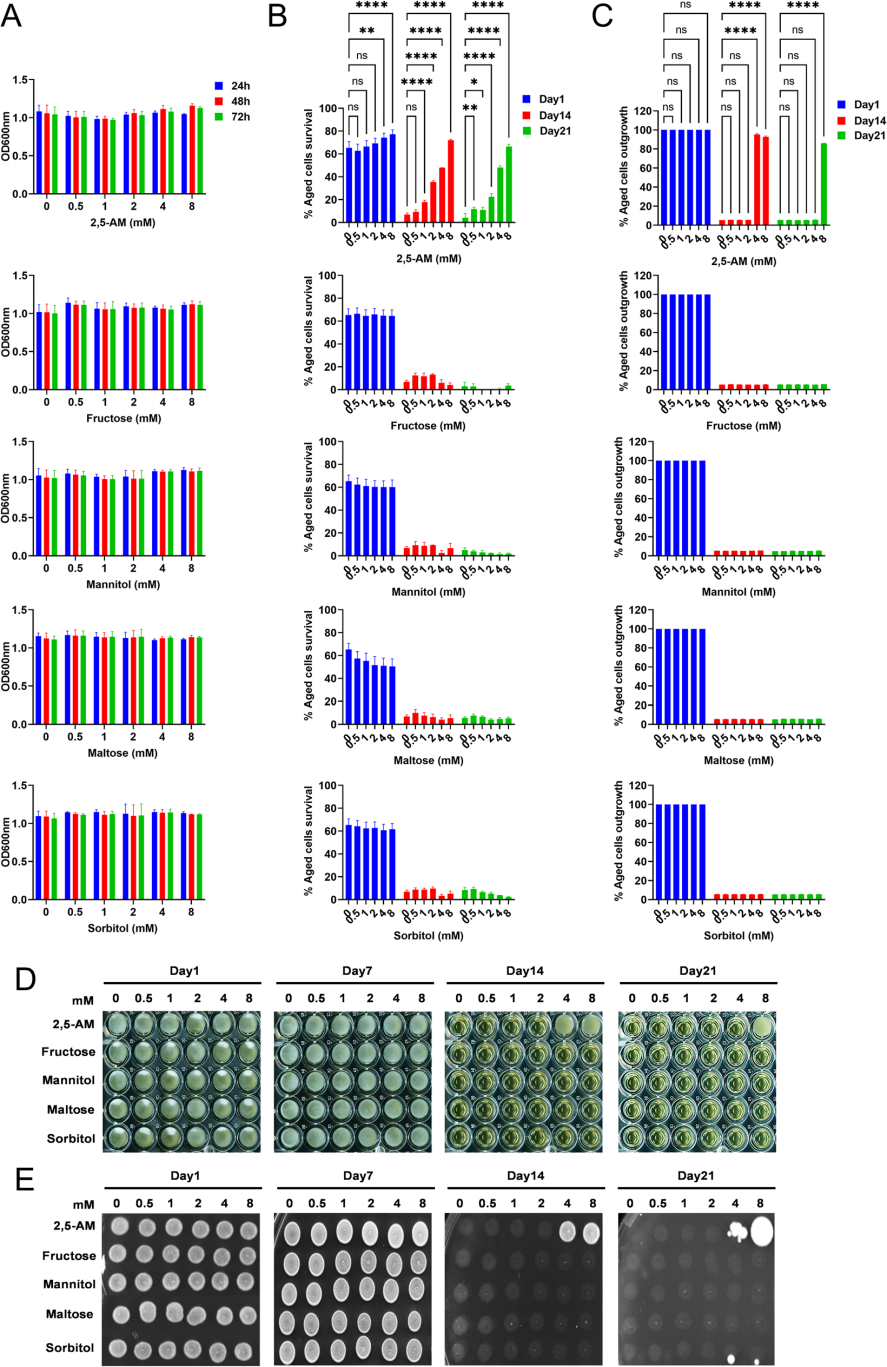
Testing the effect of 2,5-anhydro-D-mannitol analogs on chronological lifespan of the yeast. The prototrophic yeast strain (CEN.PK113-7D) was grown in the synthetic defined medium with different concentrations of 2,5-anhydro-D-mannitol (2,5-AM), D-fructose, D-mannitol, D-maltose, and D-sorbitol in 96-well plates at 30 °C. **A** Cell growth OD600nm was measured at different time points 24 h, 48 h, and 72 h using a microplate reader and graph plotted against different concentrations of 2,5-AM, fructose, mannitol, maltose, and sorbitol. **B** The chronological lifespan (CLS) of different concentrations of 2,5-AM, fructose, mannitol, maltose, and sorbitol incubated cells was determined using the propidium iodide fluorescence–based method. Cell survival at different chronological age points was quantified and the growth time point 72 h was considered as day 1. **C** The CLS of the aged cells was determined by the outgrowth method in YPD liquid medium. The growth time point 72 h was considered as day 1. At various chronological age points, a 3-μL culture were transferred to a second 96-well plate containing 200μL YPD medium. Outgrowth OD600nm in YPD liquid medium was measured after incubation for 24 h at 30 °C using a microplate reader. The graph is plotted relative to day 1. **D** Outgrowth in YPD liquid medium of 96-well plate was photographed after incubation for 24 h at 30 °C. **E** At various chronological age points, 3-μL cultures were spotted onto the YPD agar plate. Outgrowth was photographed after incubation for 48 h at 30 °C. All data represent as means ± SD.; *P < 0.05, **P < 0.01, and ****P < 0.0001 based on two-way ANOVA followed by Dunnett’s multiple comparisons test (**B** and **C**). n.s, non-significant

2,5-AM is an analog of the sugar moiety fructose [39]. To clarify whether other sugars also influence the CLS, we examined the effect of fructose, mannitol, and maltose on yeast aging. We also included sorbitol which is a non-metabolized sugar reported to increase the CLS of yeast cells by increasing the osmolarity of the culture medium [20, 45]. Firstly, we tested the effect of fructose, mannitol, maltose, and sorbitol on cell growth. Yeast cells were incubated with various concentrations of fructose, mannitol, maltose, and sorbitol similar to 2,5-AM in the SD medium. Like 2,5-AM, fructose, mannitol, maltose, and sorbitol incubated cells reached growth saturation after 24 h (Fig. 7A). Next, we measured the survival of chronologically aged cells on day 1, day 7, day 14, and day 21. Unlike 2,5-AM, fructose, mannitol, maltose, and sorbitol supplementation did not extend the lifespan of the yeast (Fig. 7B, C, D, E, and Supplementary Fig. S6). Remarkably, 2,5-AM extends the lifespan even at the late stage of chronological aging. Aged cells supplemented with 2,5-AM (8 mM) survive (~ 65%) on day 21. However, the survival of aged cells without 2,5-AM supplementation was less than 10%.

Much to our surprise, we found that sorbitol at low concentrations tested are unable to increase the CLS (Fig. 7B, C, D, E, and Supplementary Fig. S6). We hypothesized that higher sorbitol concentrations might be required to increase the CLS. We tested a range of higher concentrations including 1 M sorbitol, the concentration previously reported to affect the CLS (18% equivalent to 1 M) [20, 45]. Consistent with previous reports, we also find that very high sorbitol concentrations (between 0.5 and 1 M) increase the CLS of yeast (Supplementary Fig. S7), apparently by increasing the medium’s osmolarity. Interestingly, the CLS increase by 2,5-AM at very low concentration (2 mM) suggest its mechanism of anti-aging activity is distinct from the osmotic one. These results suggest that the anti-aging activity of 2,5-AM is specific for this sugar compound and not observed for several other, similarly structured chemicals.

## Discussion

The aging population continues to grow at an unprecedented rate worldwide. Aging is associated with a decline in cellular functions, damage accumulation, and an increasing probability of chronic diseases that lead to systems collapse and eventual death [3–7]. The prevalence of age-related pathologies late in life has a significant impact on life quality and health care costs. Thus, there is an urgent need for interventions with an effective anti-aging activity that can be exploited as a geroprotector to delay aging and prolong healthspan.

Cellular aging is controlled by a network of interconnected complex biological processes [8, 9]. Recent studies have shown that intervening in the aging biological processes can substantially increase the healthy lifespan of model organisms, including mammals [10, 14]. Chronological aging in budding yeast *Saccharomyces cerevisiae* is the well-established model system for determining the interventions of human post-mitotic cell aging [16–19].

We developed a new, fast and cheap quantitative method called PICLS for measuring CLS using (1) yeast cells incubated with media and test compounds on clear 96-well plates; (2) the fluorescent dye propidium iodide (PI), a cell impermeant that only enters dead cells [23–25]; and (3) a microplate reader for quantitative cell survival readout. To note, measuring cell density (and cell growth) at OD600nm can be performed from the same plate. For dead yeast cells on 96-well plates, we found a high linear correlation between PI fluorescence and OD600nm absorbance within an optimal cell density range corresponding to 0.05–12 ODs. This methodical approach can be utilized for quantifying cell viability in high-throughput assays.

There are several important innovations associated with PICLS: (i) PICLS is the currently only outgrowth-free method for CLS quantification. A critical time- and resource-demanding step is clipped from the protocol and, thus, PICLS is the first true high-throughput screening (HTS) for chemical agents and culturing conditions for CLS measurement. (ii) The methodical idea behind PICLS can be generally formulated as plate-based method where (a) the fraction of dead/surviving cells and (b) the total amount of cells is directly determined simultaneously from the same plate with an optical intensity measurement device (the commonly available plate reader). In this work, we used 96-well plates but 384-well plates appear also applicable (with possibly some lowered sensitivity due to the smaller amount of culture). Any coloring agent that distinguishes dead and surviving cells and that has fluorescence or absorption in a channel that does not interfere with general cell density measurement (e.g., via absorption at OD600nm) can be utilized. We applied propidium iodide that selectively marks dead cells but this is not critical for PICLS.

We note that black microplates are typically recommended for fluorescence assays because this material partially quenches the autofluorescence. However, we evaluated the method in clear microplates and found similar effectiveness as with black microplates. As black microplates are associated with high costs, this is clearly a significant advantage for large-scale high-throughput screening projects.

**Fig. 8:**
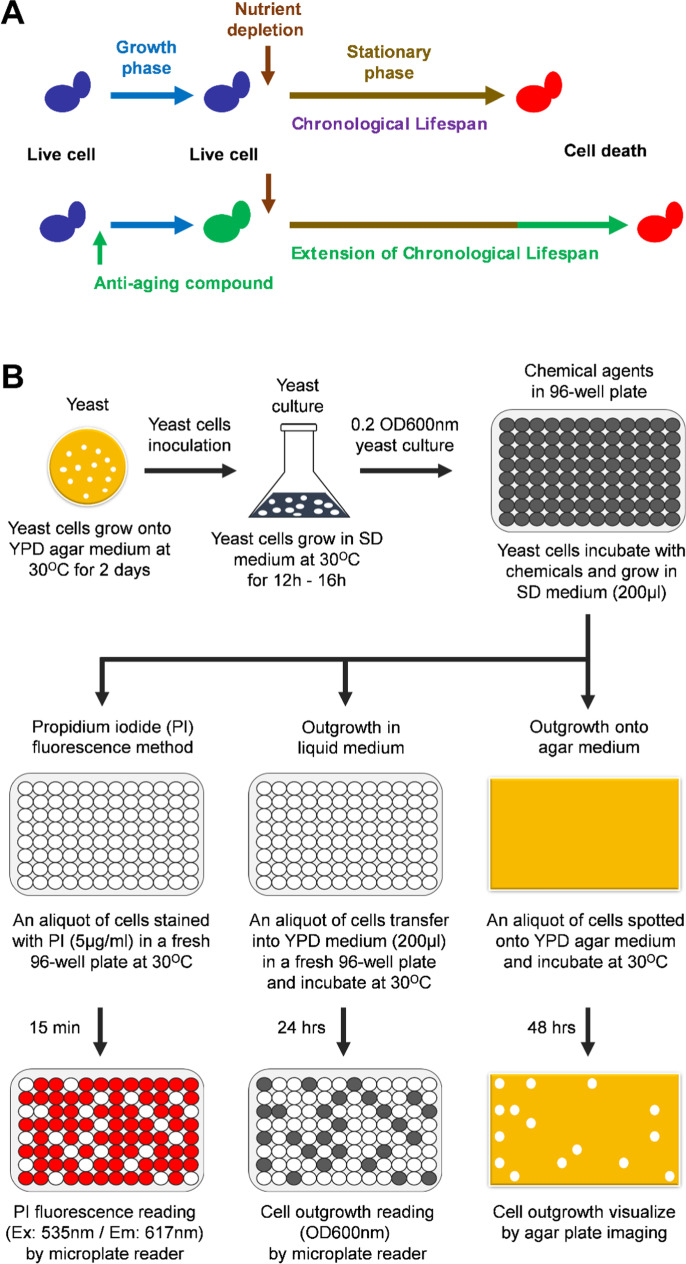
Model for identifying the anti-aging compounds and methods to measuring the chronological lifespan of the yeast. **A** Schematic representation for determining the anti-aging compounds that extend the chronological lifespan of the yeast. **B** Flowchart of propidium iodide fluorescence–based PICLS method, traditional outgrowth methods in YPD liquid medium and YPD agar medium (spotting assay) for screening the chemical agents to identify the anti-aging compounds

We validated our developed method with known anti-aging interventions such rapamycin administration and calorie restriction [10–16] by reproducing their effect on the CLS of the yeast. These results suggest that our method is effective for identifying new anti-aging interventions. We have exploited our newly developed protocol for screening chemicals agents to identify new anti-aging compounds (Fig. 8). We identified 2,5-anhydro-D-mannitol (2,5-AM) extending the CLS of the yeast. Based on our results, we suggest the usage of 2,5-AM individually or in combination with other anti-aging interventions.

2,5-AM is an analog of fructose, and both sugars can enter into the glycolytic pathway. Glycolysis is a metabolic process in which glucose is first phosphorylated by hexokinase to form glucose-6-phosphate (G6P). Phosphoglucoisomerase interconverts G6P to fructose-6-phosphate (F6P) that is further metabolized into different downstream glycolytic intermediates, including pyruvate that enters into mitochondrial TCA cycle. Like glucose, fructose is also phosphorylated by hexokinase to form F6P. Phosphofructokinase converts F6P to fructose-1,6-bisphosphate (FBP), which is further metabolized into different downstream glycolytic intermediates. 2,5-AM can also be phosphorylated by hexokinase to form 2,5-AM-6-phosphate (2,5-AM6P) [39–41]. Furthermore, phosphofructokinase converts 2,5-AM6P to 2,5-AM-1,6-bisphosphate (2,5-AMBP). However, 2,5-AMBP cannot be further metabolized into downstream glycolytic intermediates [39–41].

Since 2,5-AM is a fructose analog, we investigated whether fructose can also extend the lifespan of yeast. However, we did not observe the anti-aging activity of fructose in extending the CLS of yeast. Mannitol and maltose can also enter into glycolysis and metabolize into downstream glycolytic intermediates. We also examined the effect of mannitol and maltose on the lifespan of yeast. Like fructose, mannitol and maltose are also unable to extend the CLS of yeast.

As sorbitol, another yet non-metabolized sugar was previously reported to affect the CLS at very high concentrations (18% equivalent to 1 M) [20, 45], we wished to clarify whether the anti-aging activity of 2,5-AM might be due to the increased osmolarity of the culture medium. Our experiments showed that sorbitol fails to increase the CLS of yeast at the lower concentrations that are effective in the case of 2,5-AM. Since sorbitol requires higher concentrations to increase the CLS of yeast, the anti-aging mechanism of 2,5-AM is independent from osmolarity effects. These findings revealed that anti-aging activity of 2,5-AM is specific and not paralleled by several other analogs with similar chemical structure.

However, how 2,5-AM extends the lifespan remains to be investigated. It will be interesting to examine whether 2,5-AM directly modulates the aging hallmarks [8]. TORC1 and AMPK are the glucose-sensing complexes involved in aging process [32, 43]. However, chronological lifespan extended by inhibition of TORC1 is reduced when AMPK is inhibited. Glucose and glycolytic intermediates regulate the activity of TORC1 and AMPK [34, 46, 47]. 2,5-AMBP has been shown to inhibit the activity of aldolase that catalyzes the conversion of FBP into glyceraldehyde-3-phosphate (G3P) and dihydroxyacetone phosphate (DHAP) [39, 40]. DHAP is interconverted into G3P by triosephosphate isomerase. Recent evidence suggests that DHAP is the important glucose signal molecule that activates TORC1 [48].

Glycolytic flux is compromised when G3P and DHAP synthesis are affected, leading to less ATP production. Interestingly, AMPK is activated when the energy status of the cells is compromised and inhibits the cell growth by inhibiting the TORC1 [46, 47]. Thus, AMPK’s and TORC1’s antagonistic relation is an efficient metabolic adaptation to ensure cellular homeostasis balance for healthy cells. Although, we did not observe an effect of 2,5-AM on cell growth (Figs. 6 and 7). These findings ruled out any glucose restriction effect that compromised the cell growth (Fig. 5), however extended the lifespan with a slow-growth phenotype [49]. However, the extension of lifespan by rapamycin does not require a higher dose that compromises cell growth (Fig. 4). Therefore, we cannot exclude the possibility of anti-aging activity of 2,5-AM via TORC1/AMPK or independently of TORC1/AMPK.

Unlike the inhibitory effect of 2,5-AMBP on aldolase, it is reported to activate the pyruvate kinase, the last glycolysis enzyme [39–41]. Pyruvate kinase catalyzes the conversion of phosphoenolpyruvate to pyruvate, one of the sources for generating NAD^+^ (nicotinamide adenine dinucleotide). NAD^+^ is an important metabolite that regulates several cellular processes and functions critical for maintaining healthy cells and extending lifespan [50, 51]. Pyruvate is also the major substrate for mitochondrial reactions to generate various building block metabolites to synthesize the vital elements, including amino acids, nucleic acids, and ATP, essentially for cell survival and healthy aging [52–55]. Indeed, the decline in mitochondrial activity is associated with aging and age-related diseases [53–56]. Recently, 2,5-AM has been proposed as a potential therapeutic to treat acute myeloid leukemia (AML) cancer [57].

As a side note, we named our new method PICLS (PI-based CLS method) with the word play “pickles” in mind. Whereas all elements of our new protocol have already been described independently elsewhere, their “fermented” combination makes the measurement simple and cheap and finally high-throughput-ready.

In conclusion, we have developed a quantitative method for measuring the chronological lifespan of the yeast and identified 2,5-AM as a novel anti-aging compound. The study is currently underway to investigate the mechanisms of anti-aging activity of 2,5-AM.

## Materials and methods

### Yeast strain, media, and chemicals

The prototrophic CEN.PK113-7D strain genetic background was used in this study [35, 36]. Standard rich medium YPD (1% Bacto yeast extract, 2% Bacto peptone, and 2% glucose), YPD agar (2.5% Bacto agar), and synthetic defined (SD) medium contain 6.7 g/L yeast nitrogen base with ammonium sulfate without amino acids (DIFCO) and 2% glucose. Rapamycin (Enzo) stock solution was prepared in dimethyl sulfoxide (Sigma). The final concentration of DMSO did not exceed 1% in any assay. 2,5-Anhydro-D-mannitol (Santa Cruz), D-fructose (Sigma), D-mannitol (Sigma), D-maltose (Sigma), and D-sorbitol (Sigma) working concentrations prepared fresh by directly dissolve the powder in medium and filter sterilized.

### Yeast growth conditions

Yeast strain was recovered from frozen glycerol stock on YPD agar medium at 30 °C. Yeast was grown in SD medium overnight at 30 °C with shaking at 220 rpm. Cells grown overnight were diluted to OD600nm ~ 0.2 in fresh SD medium to initiate the chronological lifespan experiments.

### Chronological lifespan analysis

Chronological lifespan (CLS) experiments were performed in 96-well plates with a total of 200 µL yeast culture as described previously [58]. Cellular inoculum was transferred into the 96-well plate containing serially double-diluted concentrations (0–10 nM) of rapamycin. For the caloric restriction assay, cellular inoculums were prepared in SD medium containing 2%, 0.5%, and 0.25% glucose and transferred to 96-well plates. Likewise, cellular inoculum was transferred into the 96-well plates containing serially double-diluted concentrations (0–8 mM) of 2,5-anhydro-D-mannitol, D-ructose, D-mannitol, D-maltose, and D-sorbitol. Cells were incubated at 30 °C, and the growth was measured at different time points. The growth time point 72 h was considered as day 1 for the CLS assay. Cell survival was quantified at various age time points by three different approaches: (i) propidium iodide fluorescence–based method, (ii) outgrowth in YPD liquid medium, and (iii) spotting assay.

**(i) Propidium iodide fluorescence–based method:** The detailed procedures for developing the propidium iodide (PI) fluorescence–based approach are mentioned in the result section. For the CLS assay, 40-µl yeast cells on different age time points were transferred into a second 96-well plate. Cells were washed and incubated in 100 µl 1 × PBS with PI (5 µg/ml) for 15 min in the dark. Positive and negative samples control were included for quantitative analysis. Positive control (cells boiled at 100 °C for 15 min) was PI-stained and processed in the same 96-well plate. Samples without PI-stained cells were served as the negative control. After incubation, cells were washed and resuspended in 100 µl PBS. The samples fluorescence reading (excitation at 535 nm, emission at 617 nm) and OD600nm were measured by the microplate reader (BioTek). The fluorescence intensity of each sample was normalized with OD600nm. The normalized fluorescence intensity of each sample was subtracted from the background signal of unstained negative sample. The obtained fluorescence intensity of positive control sample (boiled dead cells) was considered 0% cell survival. We confirmed the cells death by allowing it to grow in the medium. Cell survival of different age time points samples were calculated by normalizing the fluorescence intensity with positive control sample (boiled cells).

Thus, cell survival is calculated via the formula:

$$\mathrm{Survival}=\left(1 - \frac{\frac{{I}_{ij}\left(535\mathrm{nm}/617\mathrm{nm}\right)}{{OD}_{ij}\left(600\mathrm{nm}\right)}-\frac{{I}_{c}\left(535\mathrm{nm}/617\mathrm{nm}\right)}{{OD}_{c}\left(600\mathrm{nm}\right)}}{\frac{{I}_{D}\left(535\mathrm{nm}/617\mathrm{nm}\right)}{{OD}_{D}\left(600\mathrm{nm}\right)}-\frac{{I}_{c}\left(535\mathrm{nm}/617\mathrm{nm}\right)}{{OD}_{c}\left(600\mathrm{nm}\right)}}\right)100\%$$

where ${I}_{ij }\left(535\mathrm{nm}/617\mathrm{nm}\right)$ is the fluorescent intensity measured at the well with plate coordinates *i* and *j* with excitation at 535 nm and emission at 617 nm, ${I}_{D} \left(535\mathrm{nm}/617\mathrm{nm}\right)$ is the fluorescent intensity measured for a cell with 100% dead cells stained with PI, ${I}_{C} \left(535\mathrm{nm}/617\mathrm{nm}\right)$ is the fluorescent intensity measured for cells without PI, and the respective OD values are the absorption measured for the respective cells at 600 nm normalizing for the amount of cells.

**(ii) Outgrowth in YPD liquid medium:** Yeast stationary culture (3-μL) of different age time points were transferred to a second 96-well plate containing 200μL YPD medium and incubated for 24 h at 30 °C. Outgrowth (OD600nm) of aged cells was measured by the microplate reader. Quantification of cell survival for each age point was determined relative to day 1 (considered 100% cells survival) as described previously [19, 20, 58].

**(iii) Spotting assay:** Yeast stationary culture (3 μL) of different age time points were spotted onto the YPD agar plate and incubated for 48 ho at 30 °C. The outgrowth of aged cells on the YPD agar plate was photographed using the BioRad GelDoc imaging system.

### Data analysis

Statistical analysis of all the results such as mean value, standard deviations, correlation (*R*^*2*^), significance and graphing were performed using GraphPad Prism v.9.3.1 software. The results were statistically compared using the ordinary one-way ANOVA and two-way ANOVA followed by multiples comparison by Dunnett’s post hoc test or Sidak’s post hoc test. In all the graph plots, *P* values are shown as *P < 0.05, **P < 0.01, ***P < 0.001, and ****P < 0.0001 were considered significant. n.s, non-significant.

## Supplementary Information

Below is the link to the electronic supplementary material.

Supplementary file1 (DOCX 2.85 MB)
