# Supplementary material for: The PICLS high-throughput screening method for agents extending cellular longevity identifies 2,5-anhydro-D-mannitol as novel anti-aging compound
Source: GeroScience. 2022 Jun 15;45(1):141–58. doi: 10.1007/s11357-022-00598-0 (PMC9886722; doi:10.1007/s11357-022-00598-0)
Supplement: Supplementary file 1 — Supplementary file1 (DOCX 2.85 MB) [file 11357_2022_598_MOESM1_ESM.docx]

**Supplementary Information**

**The PICLS high-throughput screening method for agents extending cellular longevity identifies 2,5-anhydro-D-mannitol as novel anti-aging compound**

**Authors:** Mohammad Alfatah^1*^ and Frank Eisenhaber^1,2,3*^

**Affiliations:**

1. Bioinformatics Institute (BII), A*STAR, Singapore 138671, Singapore

2. Genome Institute of Singapore (GIS), A*STAR, Singapore 138672, Singapore

3. School of Biological Sciences (SBS), Nanyang Technological University (NTU), Singapore 637551, Singapore

*To whom the correspondence should be addressed.

Email: [alfatahm@bii.a-star.edu.sg](mailto:alfatahm@bii.a-star.edu.sg) (Mohammad Alfatah)

Email: [franke@bii.a-star.edu.sg](mailto:franke@bii.a-star.edu.sg) (Frank Eisenhaber)

**Keywords**
*Saccharomyces cerevisiae*; Chronological lifespan; Chemical screening; Anti-aging compound; 2,5-anhydro-D-mannitol


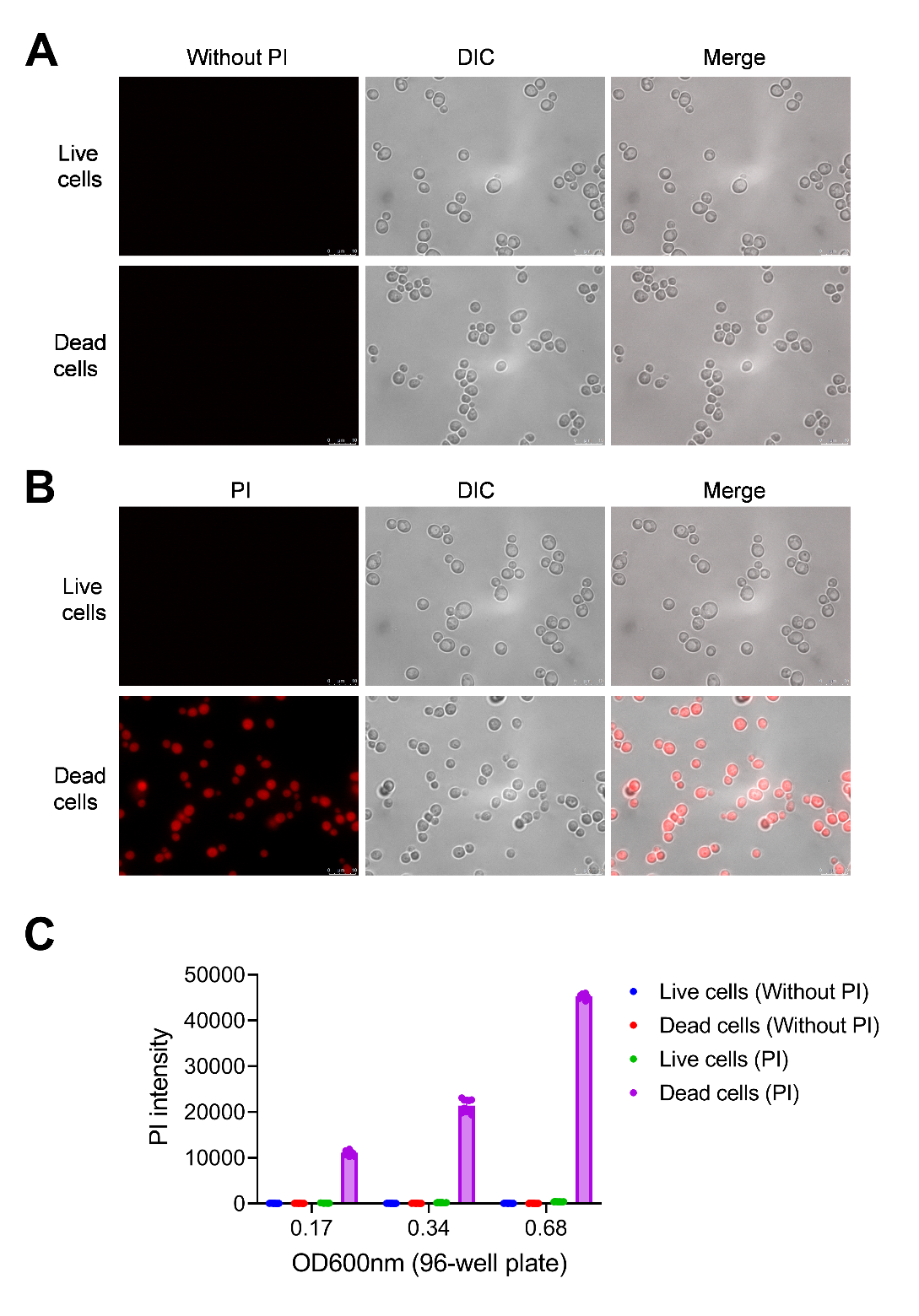


**Fig. S1. Propidium iodide staining analysis by microscopy and fluorescence intensity measuring using the microplate reader.** (A) Non-stained PI exponentially grown yeast live cells and boiled dead cells were visualized by fluorescence microscopy. (B) PI stained (5 µg/ml) exponentially grown yeast live cells and boiled dead cells were visualized by fluorescence microscopy. (C) Fluorescence intensity analysis for ten replicates of non-stained PI and stained PI live and dead cells with different OD600nm in the 96-well plate.


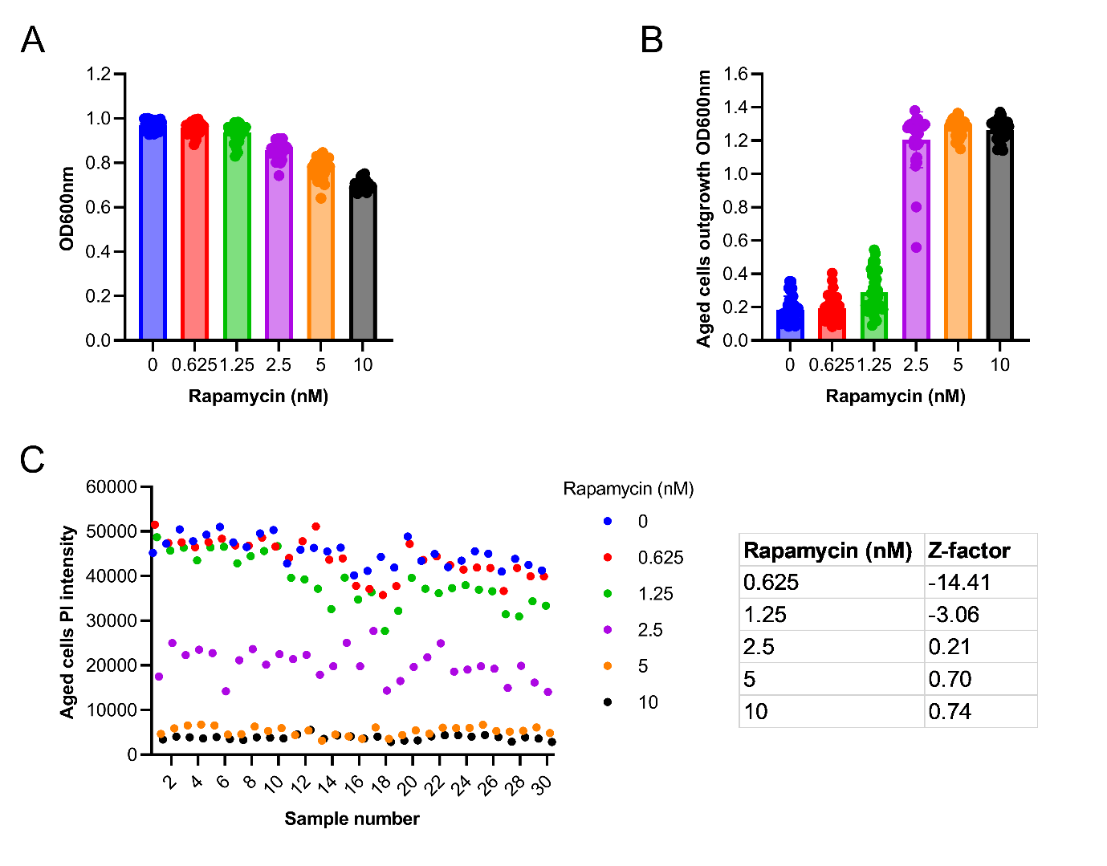


**Fig. S2. Z-factor analysis to evaluate the quality of high-throughput screening method.** The prototrophic yeast strain (CEN.PK113-7D) was grown in the synthetic defined medium with indicated concentrations of rapamycin in 96-well plates at 30 °C. For Z-factor analysis a total of 30 samples of individual rapamycin concentration was tested with DMSO control (0 nM rapamycin). (A) Cell growth OD600nm was measured at 72 h using a microplate reader and a graph is plotted against different concentrations of rapamycin. (B) The chronological lifespan (CLS) of the aged cells was determined by the outgrowth method in YPD liquid medium. The growth time point 72 h was considered as day 1. At chronological age point day 4, 3-μL cultures were transferred to a second 96-well plate containing 200μL YPD medium. Outgrowth OD600nm in YPD liquid medium was measured after incubation for 24 h at 30 ^o^C using a microplate reader. (C) The CLS of different concentrations of rapamycin incubated cells was determined using the propidium iodide fluorescence-based method. Cell survival at age point day 4 was quantified, and the growth time point 72 h was considered day 1. Z-factor of different concentrations of rapamycin was analyzed using the formula Z = 1 – [3 x (standard deviation of control sample + standard deviation of rapamycin sample) / (mean of control sample – mean of rapamycin sample)]. The Z-factor of rapamycin 5 nM and 10 nM concentrations is far above 0.5 indicating an excellent assay for the identification of anti-aging compounds that increase the CLS of the yeast.


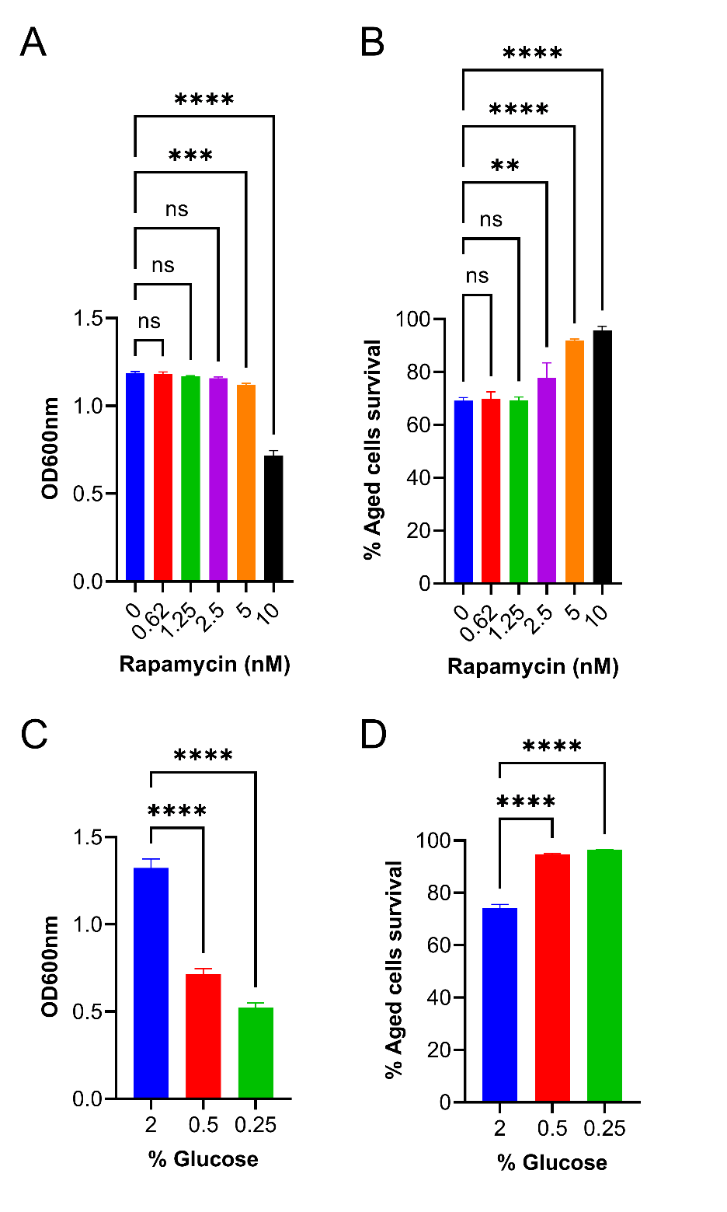


**Fig. S3. Rapamycin drug and calorie restriction extend the chronological lifespan of the yeast.** *Saccharomyces cerevisiae* BY4743 strain was grown in the synthetic defined medium supplemented with histidine (40 mg/L), leucine (160 mg/L), and uracil (40 mg/L) with different concentrations of rapamycin and under glucose restriction in 96-well plates at 30 °C. (A and C) Cell growth OD600nm was measured at 72 h using a microplate reader and graphs were plotted against indicated concentrations of rapamycin and glucose. (B and D) The chronological lifespan (CLS) of different concentrations of rapamycin and glucose incubated cells was determined using the propidium iodide fluorescence-based method. Cell survival was quantified at chronological age point day 4 and the growth time point 72 h was considered as day 1. All data represent as means ± SD.; ***P* < 0.01, and *****P* < 0.0001 based on ordinary one-way ANOVA followed by Dunnett’s multiple comparisons test. n.s: non-significant.


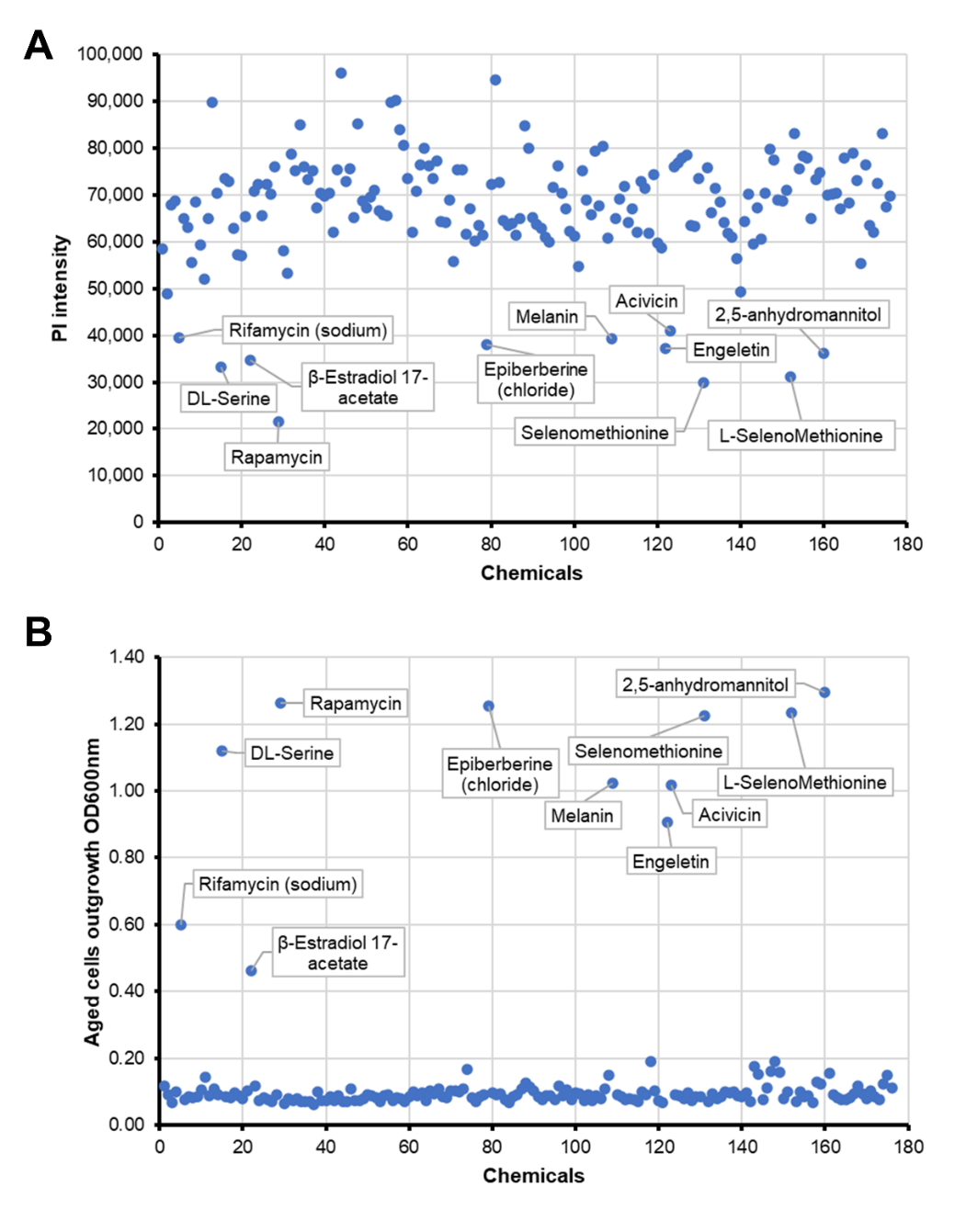


**Fig. S4. High-throughput screening of chemicals to identify novel anti-aging compounds.** The prototrophic yeast strain (CEN.PK113-7D) was grown in the synthetic defined medium with different chemicals with a single testing concentration (Supplementary Table 1) including eight replicates of DMSO and water control in 96-well plates at 30 °C. (A) The chronological lifespan (CLS) of chemical agents incubated cells was determined using the propidium iodide fluorescence-based method. Cell survival at age point day 7 was quantified, and the growth time point 72 h was considered day 1. (B) The CLS of the aged cells was determined by the outgrowth method in YPD liquid medium. The growth time point 72 h was considered as day 1. At chronological age point day 14, 3-μL cultures were transferred to a second 96-well plate containing 200μL YPD medium. Outgrowth OD600nm in YPD liquid medium was measured after incubation for 24 h at 30 ^o^C using a microplate reader.


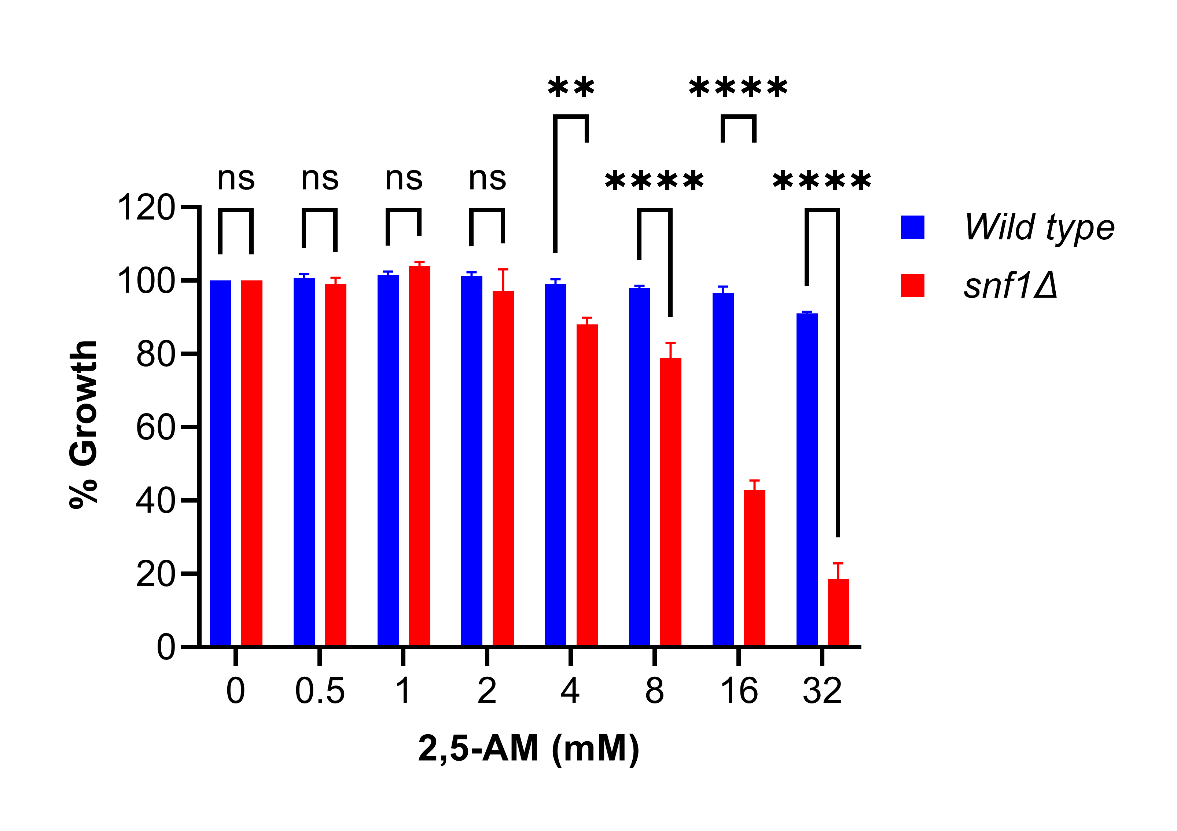


**Fig. S5. Growth of *snf1Δ* deletion strain is hypersensitive to 2,5-AM*.*** The prototrophic (CEN.PK113-7D) wild type and *snf1Δ* deletion strains were grown in the synthetic defined medium with different concentrations of 2,5-AM in 96-well plates at 30 °C. Cell growth OD600nm was measured at 72 h using a microplate reader and the graph was plotted against different concentrations of 2,5-AM. All data represent as means ± SD.; ***P* < 0.01, and *****P* < 0.0001 based on two-way ANOVA followed by Sidak’s multiple comparisons test. n.s: non-significant.


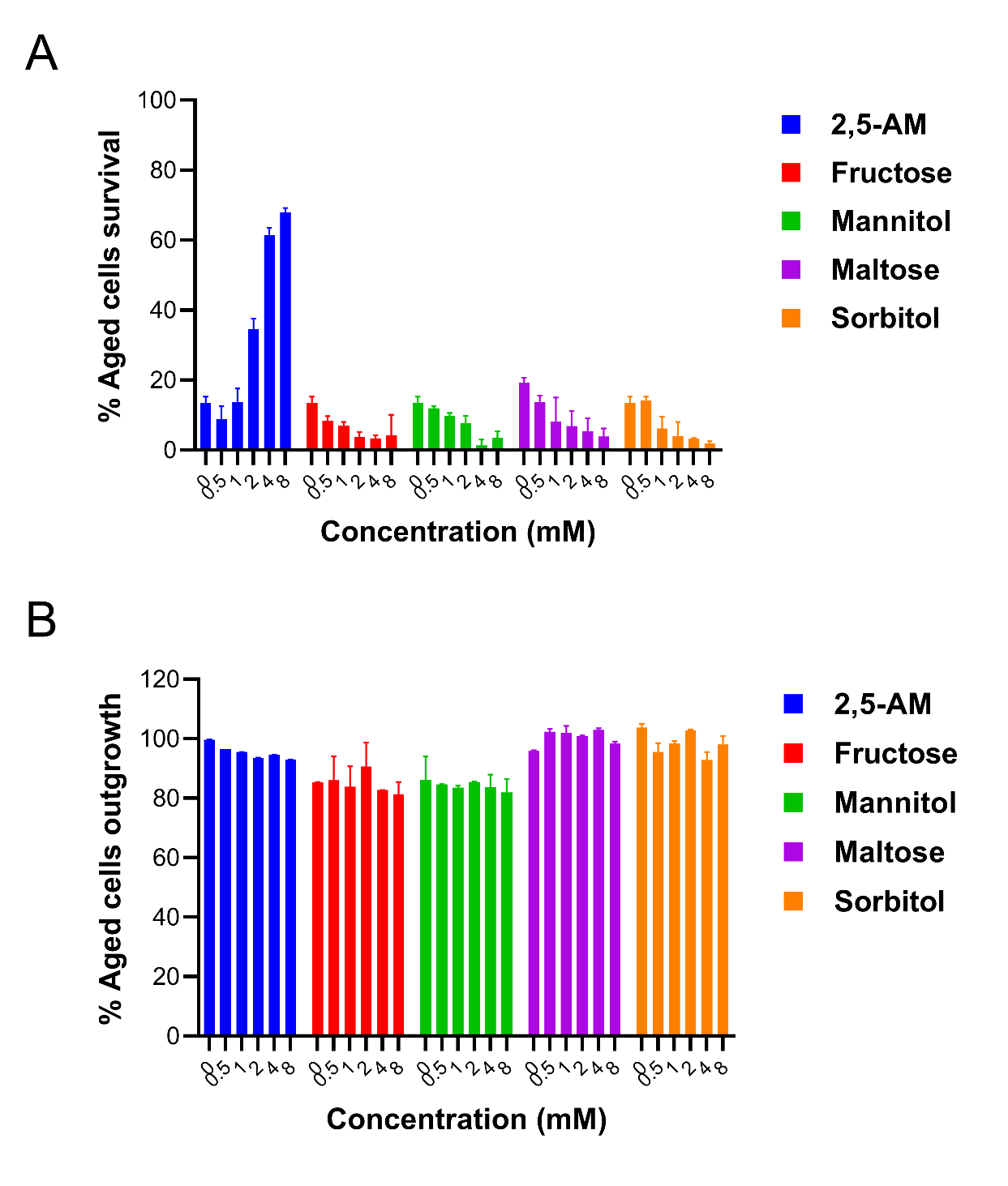


**Fig. S6. Testing the effect of 2,5-anhydro-D-mannitol analogs on chronological lifespan of the yeast.** The prototrophic yeast strain (CEN.PK113-7D) was grown in the synthetic defined medium with different concentrations of 2,5-anhydro-D-mannitol (2,5-AM), D-Fructose, D-Mannitol, D-Maltose and D-Sorbitol in 96-well plates at 30 °C. (A) The chronological lifespan (CLS) of different concentrations of 2,5-AM, fructose, mannitol, maltose and sorbitol incubated cells was determined using the propidium iodide fluorescence-based method. Cell survival was quantified at chronological age point day7 and the growth time point 72 h was considered as day 1. (B) The CLS of the aged cells was determined by the outgrowth method in YPD liquid medium. The growth time point 72 h was considered as day 1. At chronological age point day 7, 3-μL cultures were transferred to a second 96-well plate containing 200μL YPD medium. Outgrowth OD600nm in YPD liquid medium was measured after incubation for 24 h at 30 ^o^C using a microplate reader. The graph is plotted relative to day 1.


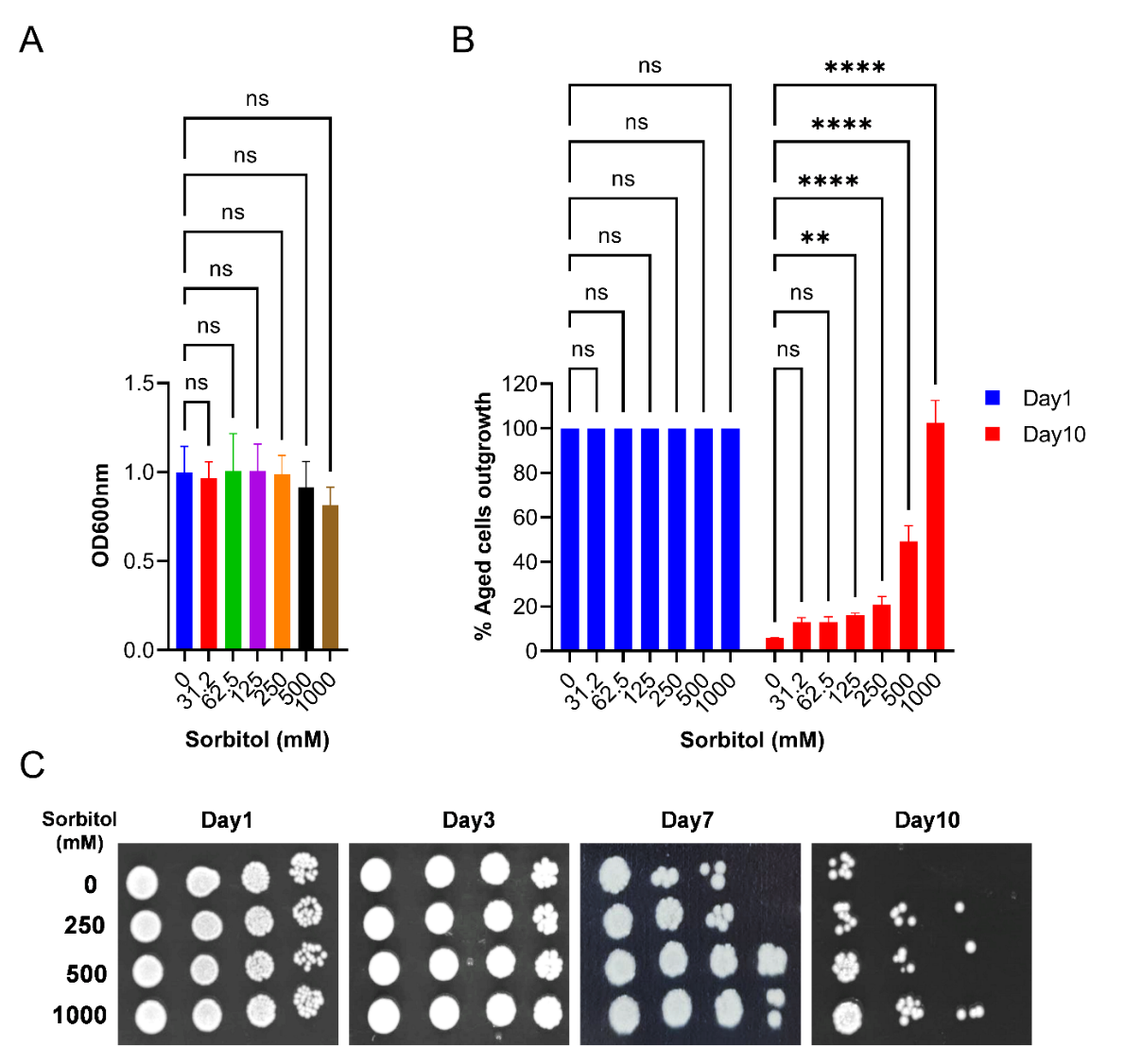


**Fig. S7. Sorbitol extends the chronological lifespan of the yeast.** The prototrophic yeast strain (CEN.PK113-7D) was grown in the synthetic defined medium with different concentrations of sorbitol in 96-well plates at 30 °C. (A) Cell growth OD600nm was measured at 72 h using a microplate reader and a graph was plotted against different concentrations of sorbitol. (B) The chronological lifespan (CLS) of the aged cells was determined by the outgrowth method in YPD liquid medium. The growth time point 72 h was considered as day 1. At chronological age points day 1 and day 4, 3-μL cultures were transferred to a second 96-well plate containing 200μL YPD medium. Outgrowth OD600nm in YPD liquid medium was measured after incubation for 24 h at 30 ^o^C using a microplate reader. The outgrowth is plotted relative to day 1. (C) At various chronological age points, 3-μL cultures of serial 10-fold dilutions were spotted onto the YPD agar plate. Outgrowth was photographed after incubation for 48 h at 30 ^o^C. All data represent as means ± SD.; ***P* < 0.01, and *****P* < 0.0001 based on ordinary one-way ANOVA (A) and two-way ANOVA (B) followed by Dunnett’s multiple comparisons test. n.s: non-significant.

**Supplementary Table 1**

List of chemicals screened in this study.

| **Number** | **Chemical name** | **Solvent** | **Concentration tested** | **PI intensity** | **Outgrowth** | **Yeast CLS increase** |
| --- | --- | --- | --- | --- | --- | --- |
| 1 | Aucubin | DMSO | 100µM | 58,414.0 | 0.1 | No |
| 2 | D-Glucuronic acid | DMSO | 100µM | 48,839.6 | 0.1 | No |
| 3 | β-Lapachone | DMSO | 100µM | 67,892.9 | 0.1 | No |
| 4 | Isocytosine | DMSO | 100µM | 68,813.6 | 0.1 | No |
| 5 | Rifamycin (sodium) | DMSO | 100µM | 39,446.0 | 0.6 | Yes |
| 6 | Cryptochlorogenic acid | DMSO | 100µM | 65,040.0 | 0.1 | No |
| 7 | Isochlorogenic acid A | DMSO | 100µM | 63,157.7 | 0.1 | No |
| 8 | Rhynchophylline | DMSO | 100µM | 55,562.5 | 0.1 | No |
| 9 | Mupirocin | DMSO | 100µM | 68,477.7 | 0.1 | No |
| 10 | 3,5-Dihydroxybenzoic acid | DMSO | 100µM | 59,369.7 | 0.1 | No |
| 11 | Ethylparaben | DMSO | 100µM | 52,097.7 | 0.1 | No |
| 12 | Nudifloramide | DMSO | 100µM | 65,023.3 | 0.1 | No |
| 13 | 4-Methoxycinnamic acid | DMSO | 100µM | 89,890.5 | 0.1 | No |
| 14 | Cefoselis (sulfate) | DMSO | 100µM | 70,425.6 | 0.1 | No |
| 15 | DL-Serine | DMSO | 100µM | 33,155.7 | 1.1 | Yes |
| 16 | Avibactam (sodium) | DMSO | 100µM | 73,562.3 | 0.1 | No |
| 17 | (S)-2-Hydroxy-3-phenylpropanoic acid | DMSO | 100µM | 72,969.5 | 0.1 | No |
| 18 | Vincristine (sulfate) | DMSO | 100µM | 62,812.1 | 0.1 | No |
| 19 | N-Acetyl-L-tyrosine | DMSO | 100µM | 57,322.5 | 0.1 | No |
| 20 | Rubitecan | DMSO | 100µM | 57,075.3 | 0.1 | No |
| 21 | Royal Jelly acid | DMSO | 100µM | 65,406.1 | 0.1 | No |
| 22 | β-Estradiol 17-acetate | DMSO | 100µM | 34,621.0 | 0.5 | Yes |
| 23 | 3,4-Dimethoxycinnamic acid | DMSO | 100µM | 70,914.2 | 0.1 | No |
| 24 | 2-Naphthol | DMSO | 100µM | 72,298.9 | 0.1 | No |
| 25 | N2,N2-Dimethylguanosine | DMSO | 100µM | 65,619.9 | 0.1 | No |
| 26 | Proxyphylline | DMSO | 100µM | 72,232.1 | 0.1 | No |
| 27 | Amarogentin | DMSO | 100µM | 70,083.5 | 0.1 | No |
| 28 | Lappaconitine (hydrobromide) | DMSO | 100µM | 76,000.0 | 0.1 | No |
| 29 | Rapamycin | DMSO | 100µM | 21,460.5 | 1.3 | Yes |
| 30 | 4-Ethylphenol | DMSO | 100µM | 58,041.7 | 0.1 | No |
| 31 | Purine | DMSO | 100µM | 53,185.1 | 0.1 | No |
| 32 | (S)-Indoximod | DMSO | 100µM | 78,648.2 | 0.1 | No |
| 33 | Actinonin | DMSO | 100µM | 75,254.2 | 0.1 | No |
| 34 | Cirsimaritin | DMSO | 100µM | 85,017.1 | 0.1 | No |
| 35 | 6-Biopterin | DMSO | 100µM | 75,974.5 | 0.1 | No |
| 36 | Hederagenin | DMSO | 100µM | 73,281.0 | 0.1 | No |
| 37 | Pentadecanoic acid | DMSO | 100µM | 75,173.9 | 0.1 | No |
| 38 | Staurosporine | DMSO | 100µM | 67,250.9 | 0.1 | No |
| 39 | Xanthoxylin | DMSO | 100µM | 70,352.1 | 0.1 | No |
| 40 | D-(+)-Cellobiose | DMSO | 100µM | 69,857.4 | 0.1 | No |
| 41 | Escin | DMSO | 100µM | 70,495.1 | 0.1 | No |
| 42 | 5α-Cholestan-3-one | DMSO | 100µM | 62,022.6 | 0.1 | No |
| 43 | 20(S)-Hydroxycholesterol | DMSO | 100µM | 75,450.9 | 0.1 | No |
| 44 | Anthraquinone-2-carboxylic acid | DMSO | 100µM | 96,099.2 | 0.1 | No |
| 45 | L-Kynurenine | DMSO | 100µM | 72,824.1 | 0.1 | No |
| 46 | Quinine (hydrochloride dihydrate) | DMSO | 100µM | 75,648.7 | 0.1 | No |
| 47 | DL-3-Phenyllactic acid | DMSO | 100µM | 65,266.9 | 0.1 | No |
| 48 | Retinoic acid | DMSO | 100µM | 85,242.6 | 0.1 | No |
| 49 | Schisantherin B | DMSO | 100µM | 68,765.7 | 0.1 | No |
| 50 | Narciclasine | DMSO | 100µM | 67,161.8 | 0.1 | No |
| 51 | Doripenem (monohydrate) | DMSO | 100µM | 69,608.7 | 0.1 | No |
| 52 | Diflorasone | DMSO | 100µM | 71,051.3 | 0.1 | No |
| 53 | Chlorogenic acid | DMSO | 100µM | 66,593.5 | 0.1 | No |
| 54 | Glucosamine (hydrochloride) | DMSO | 100µM | 65,905.5 | 0.1 | No |
| 55 | Gardenoside | DMSO | 100µM | 65,525.5 | 0.1 | No |
| 56 | Aloin B | DMSO | 100µM | 89,712.0 | 0.1 | No |
| 57 | 4-Methylumbelliferone | DMSO | 100µM | 90,234.6 | 0.1 | No |
| 58 | Lanolin | DMSO | 100µM | 84,056.6 | 0.1 | No |
| 59 | (R)-pyrrolidine-2-carboxylic acid | DMSO | 100µM | 80,551.7 | 0.1 | No |
| 60 | Glycodeoxycholic acid (monohydrate) | DMSO | 100µM | 73,459.3 | 0.1 | No |
| 61 | (-)-Epigallocatechin | DMSO | 100µM | 62,025.6 | 0.1 | No |
| 62 | 2'-Deoxyadenosine | DMSO | 100µM | 70,796.4 | 0.1 | No |
| 63 | Wogonin | DMSO | 100µM | 76,348.2 | 0.1 | No |
| 64 | Schisandrol B | DMSO | 100µM | 80,051.7 | 0.1 | No |
| 65 | 2,4-Dihydroxybenzoic acid | DMSO | 100µM | 76,326.3 | 0.1 | No |
| 66 | Hederacoside C | DMSO | 100µM | 73,515.9 | 0.1 | No |
| 67 | Noricaritin | DMSO | 100µM | 77,179.8 | 0.1 | No |
| 68 | Canrenone | DMSO | 100µM | 64,295.2 | 0.1 | No |
| 69 | Glycoursodeoxycholic acid | DMSO | 100µM | 64,205.2 | 0.1 | No |
| 70 | Glycodeoxycholic Acid | DMSO | 100µM | 68,981.6 | 0.1 | No |
| 71 | Cilastatin | DMSO | 100µM | 55,728.9 | 0.1 | No |
| 72 | Emetine (dihydrochloride hydrate) | DMSO | 100µM | 75,341.5 | 0.1 | No |
| 73 | Norgestrel | DMSO | 100µM | 75,338.8 | 0.1 | No |
| 74 | Heterophyllin B | DMSO | 100µM | 61,587.8 | 0.2 | No |
| 75 | Hispidin | DMSO | 100µM | 67,098.8 | 0.1 | No |
| 76 | Cinnamic acid | DMSO | 100µM | 60,256.3 | 0.1 | No |
| 77 | Catechin | DMSO | 100µM | 63,589.5 | 0.1 | No |
| 78 | [6]-Gingerol | DMSO | 100µM | 61,395.3 | 0.1 | No |
| 79 | Epiberberine (chloride) | DMSO | 100µM | 38,027.2 | 1.3 | Yes |
| 80 | Terconazole | DMSO | 100µM | 72,350.0 | 0.1 | No |
| 81 | Isoalantolactone | DMSO | 100µM | 94,566.8 | 0.1 | No |
| 82 | 2-Furoic acid | DMSO | 100µM | 72,748.2 | 0.1 | No |
| 83 | Vanillic acid | DMSO | 100µM | 64,546.4 | 0.1 | No |
| 84 | 5-Acetylsalicylic acid | DMSO | 100µM | 63,538.6 | 0.1 | No |
| 85 | Guggulsterone | DMSO | 100µM | 63,881.9 | 0.1 | No |
| 86 | Thymol | DMSO | 100µM | 61,503.8 | 0.1 | No |
| 87 | Homovanillic acid | DMSO | 100µM | 65,033.1 | 0.1 | No |
| 88 | Uridine | DMSO | 100µM | 84,894.9 | 0.1 | No |
| 89 | Gramicidin | DMSO | 100µM | 79,922.3 | 0.1 | No |
| 90 | Gossypol | DMSO | 100µM | 65,220.1 | 0.1 | No |
| 91 | Cyanidin-3-O-galactoside (chloride) | DMSO | 100µM | 63,761.1 | 0.1 | No |
| 92 | Kaempferol 3-O-β-D-glucuronide | DMSO | 100µM | 62,874.6 | 0.1 | No |
| 93 | L-Homoserine | DMSO | 100µM | 60,922.1 | 0.1 | No |
| 94 | D-Ribose(mixture of isomers) | DMSO | 100µM | 60,023.1 | 0.1 | No |
| 95 | Harmane | DMSO | 100µM | 71,690.9 | 0.1 | No |
| 96 | Geraniol | DMSO | 100µM | 76,340.1 | 0.1 | No |
| 97 | 3,4-Dimethoxyphenylacetic acid | DMSO | 100µM | 70,394.7 | 0.1 | No |
| 98 | 2-Phenylpropionic acid | DMSO | 100µM | 66,998.4 | 0.1 | No |
| 99 | Deltonin | DMSO | 100µM | 62,357.6 | 0.1 | No |
| 100 | 2-Oxovaleric acid | DMSO | 100µM | 61,246.4 | 0.1 | No |
| 101 | Estrone sulfate (potassium) | DMSO | 100µM | 54,751.8 | 0.1 | No |
| 102 | (R)-Citronellol | DMSO | 100µM | 75,290.6 | 0.1 | No |
| 103 | Euphorbia Factor L2 | DMSO | 100µM | 68,871.8 | 0.1 | No |
| 104 | β-Aminopropionitrile | DMSO | 100µM | 65,865.6 | 0.1 | No |
| 105 | Bronopol | DMSO | 100µM | 79,284.9 | 0.1 | No |
| 106 | Lincomycin (hydrochloride monohydrate) | DMSO | 100µM | 67,751.3 | 0.1 | No |
| 107 | Tridecanedioic acid | DMSO | 100µM | 80,407.7 | 0.1 | No |
| 108 | Calcifediol | DMSO | 100µM | 60,747.4 | 0.2 | No |
| 109 | Melanin | DMSO | 100µM | 39,360.1 | 1.0 | Yes |
| 110 | Methyl acetylacetate | DMSO | 100µM | 64,956.3 | 0.1 | No |
| 111 | L-Cysteine (hydrochloride hydrate) | DMSO | 100µM | 69,216.4 | 0.1 | No |
| 112 | Impulsin | DMSO | 100µM | 71,903.7 | 0.1 | No |
| 113 | Formamide | DMSO | 100µM | 64,135.2 | 0.1 | No |
| 114 | 3-Chloro-L-tyrosine | DMSO | 100µM | 67,122.0 | 0.1 | No |
| 115 | Tetramethylpyrazine | DMSO | 100µM | 62,003.6 | 0.1 | No |
| 116 | Esculin | DMSO | 100µM | 72,890.7 | 0.1 | No |
| 117 | DL-Panthenol | DMSO | 100µM | 71,468.6 | 0.1 | No |
| 118 | Baimaside | DMSO | 100µM | 61,895.7 | 0.2 | No |
| 119 | Benzyl acetate | DMSO | 100µM | 74,321.9 | 0.1 | No |
| 120 | Gentiopicroside | DMSO | 100µM | 59,804.7 | 0.1 | No |
| 121 | Guaiazulene | DMSO | 100µM | 58,776.1 | 0.1 | No |
| 122 | Engeletin | DMSO | 100µM | 37,144.4 | 0.9 | Yes |
| 123 | Acivicin | DMSO | 100µM | 41,066.2 | 1.0 | Yes |
| 124 | Tenofovir (hydrate) | Water | 100µM | 76,065.0 | 0.1 | No |
| 125 | Metyrosine | Water | 100µM | 76,947.2 | 0.1 | No |
| 126 | Ripasudil | Water | 100µM | 77,962.0 | 0.1 | No |
| 127 | Diphenmanil (methylsulfate) | Water | 100µM | 78,623.4 | 0.1 | No |
| 128 | Ambroxol (hydrochloride) | Water | 100µM | 63,509.0 | 0.1 | No |
| 129 | VAL-083 | Water | 100µM | 63,325.5 | 0.1 | No |
| 130 | Aliskiren (hemifumarate) | Water | 100µM | 73,607.3 | 0.1 | No |
| 131 | Selenomethionine | Water | 100µM | 29,837.8 | 1.2 | Yes |
| 132 | Cisatracurium (besylate) | Water | 100µM | 75,862.3 | 0.1 | No |
| 133 | DL-Methionine methylsulfonium (chloride) | Water | 100µM | 66,281.7 | 0.1 | No |
| 134 | Biapenem | Water | 100µM | 71,409.3 | 0.1 | No |
| 135 | Dipotassium glycyrrhizinate | Water | 100µM | 68,519.8 | 0.1 | No |
| 136 | L-Ornithine (hydrochloride) | Water | 100µM | 64,179.1 | 0.1 | No |
| 137 | Fosphenytoin (disodium) | Water | 100µM | 61,860.0 | 0.1 | No |
| 138 | (R)-Baclofen | Water | 100µM | 61,049.3 | 0.1 | No |
| 139 | Trapidil | Water | 100µM | 56,443.9 | 0.1 | No |
| 140 | Xanthinol Nicotinate | Water | 100µM | 49,233.6 | 0.1 | No |
| 141 | Cerivastatin (sodium) | Water | 100µM | 64,250.5 | 0.1 | No |
| 142 | Aprotinin | Water | 100µM | 70,144.6 | 0.1 | No |
| 143 | Flavin adenine dinucleotide (disodium salt) | Water | 100µM | 59,508.7 | 0.2 | No |
| 144 | Biperiden (Hydrochloride) | Water | 100µM | 67,178.1 | 0.2 | No |
| 145 | Streptomycin (sulfate) | Water | 100µM | 60,638.5 | 0.1 | No |
| 146 | Cefadroxil | Water | 100µM | 70,326.1 | 0.1 | No |
| 147 | Diclofenac (Sodium) | Water | 100µM | 79,704.3 | 0.2 | No |
| 148 | Estramustine (phosphate sodium) | Water | 100µM | 77,514.0 | 0.2 | No |
| 149 | Amifostine | Water | 100µM | 69,013.2 | 0.2 | No |
| 150 | Quetiapine sulfoxide (dihydrochloride) | Water | 100µM | 68,761.6 | 0.1 | No |
| 151 | Minocycline (hydrochloride) | Water | 100µM | 71,078.2 | 0.1 | No |
| 152 | L-SelenoMethionine | Water | 100µM | 31,240.0 | 1.2 | Yes |
| 153 | Cangrelor (tetrasodium) | Water | 100µM | 83,173.6 | 0.1 | No |
| 154 | Chondroitin (sulfate) | Water | 30µg/ml | 75,572.6 | 0.1 | No |
| 155 | Hyaluronidase | Water | 30µg/ml | 78,406.7 | 0.1 | No |
| 156 | Heparin (Lithium salt) | Water | 30µg/ml | 77,829.1 | 0.1 | No |
| 157 | Heparin (sodium salt) (MW 15kDa) | Water | 30µg/ml | 64,969.0 | 0.1 | No |
| 158 | Gentamicin (sulfate) | Water | 30µg/ml | 73,296.2 | 0.1 | No |
| 159 | Polidocanol | Water | 30µg/ml | 74,745.5 | 0.1 | No |
| 160 | 2,5-anhydromannitol | Water | 300µg/ml | 36,240.0 | 1.3 | Yes |
| 161 | DMSO control 1 | DMSO |  | 69,929.1 | 0.2 |  |
| 162 | DMSO control 2 | DMSO |  | 70,278.0 | 0.1 |  |
| 163 | DMSO control 3 | DMSO |  | 70,494.8 | 0.1 |  |
| 164 | DMSO control 4 | DMSO |  | 67,035.1 | 0.1 |  |
| 165 | DMSO control 5 | DMSO |  | 78,011.0 | 0.1 |  |
| 166 | DMSO control 6 | DMSO |  | 68,377.5 | 0.1 |  |
| 167 | DMSO control 7 | DMSO |  | 78,899.7 | 0.1 |  |
| 168 | DMSO control 8 | DMSO |  | 73,013.1 | 0.1 |  |
| 169 | Water control 1 | Water |  | 55,358.7 | 0.1 |  |
| 170 | Water control 2 | Water |  | 76,405.5 | 0.1 |  |
| 171 | Water control 3 | Water |  | 63,579.4 | 0.1 |  |
| 172 | Water control 4 | Water |  | 62,094.3 | 0.1 |  |
| 173 | Water control 5 | Water |  | 72,575.8 | 0.1 |  |
| 174 | Water control 6 | Water |  | 83,192.6 | 0.1 |  |
| 175 | Water control 7 | Water |  | 67,575.6 | 0.2 |  |
| 176 | Water control 8 | Water |  | 69,669.4 | 0.1 |  |
